# Metabolic Rewiring by Pomegranate Juice and Punicalagin Is Associated with NHR-49/MDT-15-Related Responses and Enhanced Stress Resilience

**DOI:** 10.3390/ijms27219852

**Published:** 2026-11-03

**Authors:** Monika N. Todorova, Biser K. Binev, Christina Ploumi, Eleni Tsakiri, Antonis Roussos, Dimitre A. Ivanov, Martina S. Savova, Konstantinos Palikaras, Milen I. Georgiev

**Affiliations:** 1 Laboratory of Metabolomics, Institute of Microbiology, Bulgarian Academy of Sciences, 4000 Plovdiv, Bulgaria; mntodorova@yahoo.com (M.N.T.); biser.binev@abv.bg (B.K.B.); milengeorgiev@gbg.bg (M.I.G.); 2 Department of Plant Cell Biotechnology, Center of Plant Systems Biology and Biotechnology, 4023 Plovdiv, Bulgaria; 3 Department of Physiology, Medical School, National and Kapodistrian University of Athens, 115 27 Athens, Greece; cploumi@med.uoa.gr (C.P.); eltsakiri@auth.gr (E.T.); antonisrous@med.uoa.gr (A.R.); palikarask@med.uoa.gr (K.P.); 4 Department of Plant Metabolomics, Center of Plant Systems Biology and Biotechnology, 4023 Plovdiv, Bulgaria; d.ivanov@cpsbb.eu; 5 Department of Biology, University of Western Ontario, London, ON N6A 5B7, Canada

**Keywords:** *Punica granatum*, pomegranate, punicalagin, metabolism, healthspan, *Caenorhabditis elegans*

## Abstract

Metabolic homeostasis is a key determinant of stress resilience and healthy ageing. Pomegranate juice (PGJ) and its characteristic ellagitannin punicalagin (PUN) have been associated with beneficial metabolic effects, but the mechanisms through which they influence metabolic adaptation and healthspan, and whether they act through shared or distinct pathways, remain poorly defined. We investigated the effects of lyophilized PGJ (50, 100, and 200 μg/mL) and PUN (25, 50, and 100 μM) on metabolic regulation and stress resilience in *Caenorhabditis elegans*. Nuclear magnetic resonance spectroscopy was employed for metabolic profiling of PGJ, whereas ultra-performance liquid chromatography-quadrupole time-of-flight mass spectrometry enabled the quantification of PUN at 2.4 ± 0.127 µg/mg lyophilized juice. Under glucose-enriched conditions, PGJ (200 μg/mL) and PUN (100 μM) reduced normalised Nile Red fluorescence to 0.53 and 0.59 of vehicle levels. On day 10 of adulthood, both treatments increased mean survival under oxidative stress by 1.73- and 1.50-fold, respectively. Both treatments increased MDT-15 reporter response (1.13- and 1.44-fold, respectively), whereas PGJ preferentially increased AAK-2- and FAT-6-associated responses and PUN elevated NHR-49- and FAT-7 expression. PUN also improved locomotor performance under amyloid-β-induced proteotoxic stress. Overall, PGJ and PUN elicited partially overlapping metabolic responses in *C. elegans*, with NHR-49 and MDT-15 emerging as treatment-responsive regulators associated with lipid homeostasis.

## 1. Introduction

Loss of metabolic homeostasis is a prominent feature of ageing and contributes to reduced stress resilience and functional decline. Conserved nutrient- and lipid-sensing pathways therefore represent potential targets for interventions aimed at preserving healthspan. Pomegranate (*Punica granatum* L., Punicaceae) is a widely consumed fruit whose juice contains phenolic acids, flavonoids, anthocyanins, tannins, and ellagitannins. Among these constituents, punicalagin (PUN) has reported antioxidant, anti-inflammatory, and metabolic regulatory activities and may contribute to the biological effects of pomegranate products [1,2,3,4,5].

Dysregulated lipid metabolism accompanies multiple age-associated disorders, including atherosclerosis, obesity, and neurodegenerative diseases [6,7,8]. Metabolic dysfunction contributes to oxidative stress and chronic inflammation, and can compromise physiological resilience and functional capacity during ageing [9,10]. Accordingly, the maintenance of metabolic homeostasis represents an important determinant for healthy ageing.

Many regulators of lipid homeostasis and metabolic adaptation in mammals, including AMP-activated protein kinase (AMPK), nuclear hormone receptors, and Mediator-associated transcriptional regulators, are evolutionarily conserved or have functional orthologues in the nematode *Caenorhabditis elegans* [11,12]. In particular, the AMPK orthologue AAK-2 and the nuclear hormone receptor NHR-49, together with the Mediator component MDT-15, play central roles in coordinating lipid metabolism and metabolic adaptation in *C. elegans* [12,13].

Although pomegranate juice and PUN have been associated with beneficial effects on metabolic plasticity and healthspan [6,14,15,16,17], the molecular basis of these effects remains poorly defined. It is therefore unclear how the complex juice matrix and its major ellagitannin PUN influence metabolic regulation and healthspan, and whether they engage common or distinct regulatory mechanisms linked to lipid homeostasis and metabolic adaptation.

To address this mechanistic gap, we investigated the biological effects of lyophilized pomegranate juice (PGJ) and independently examined punicalagin (PUN), a characteristic ellagitannin identified in the investigated juice preparation in *C. elegans*. Rather than establishing a concentration-matched comparison or determining the contribution of PUN to the overall activity of PGJ, our objective was to characterise the metabolic and stress-related responses associated with the complex juice matrix and the selected individual constituent, with particular emphasis on conserved molecular regulators of lipid homeostasis and metabolic adaptation. The PGJ was first chemically characterised by nuclear magnetic resonance (NMR) spectroscopy and Ultra-performance liquid chromatography coupled to quadrupole time-of-flight mass spectrometry (UPLC-qToF-MS). We then evaluated the effects of PGJ and PUN across stress-resilience, locomotor, lipid homeostasis, and mitochondrial parameters under basal and glucose-induced metabolic stress. To further investigate the molecular pathways underlying these effects, genetic mutant analysis, GFP-labelled reporter strains, and gene expression analyses were employed. Finally, PUN was examined under chronic proteotoxic stress in the GMC101 model. We tested whether PGJ and PUN converge on shared conserved metabolic regulators while eliciting distinct downstream responses associated with functional healthspan.

## 2. Results

### 2.1. Metabolite Profiling Based on NMR Spectroscopy

**Figure 1:**
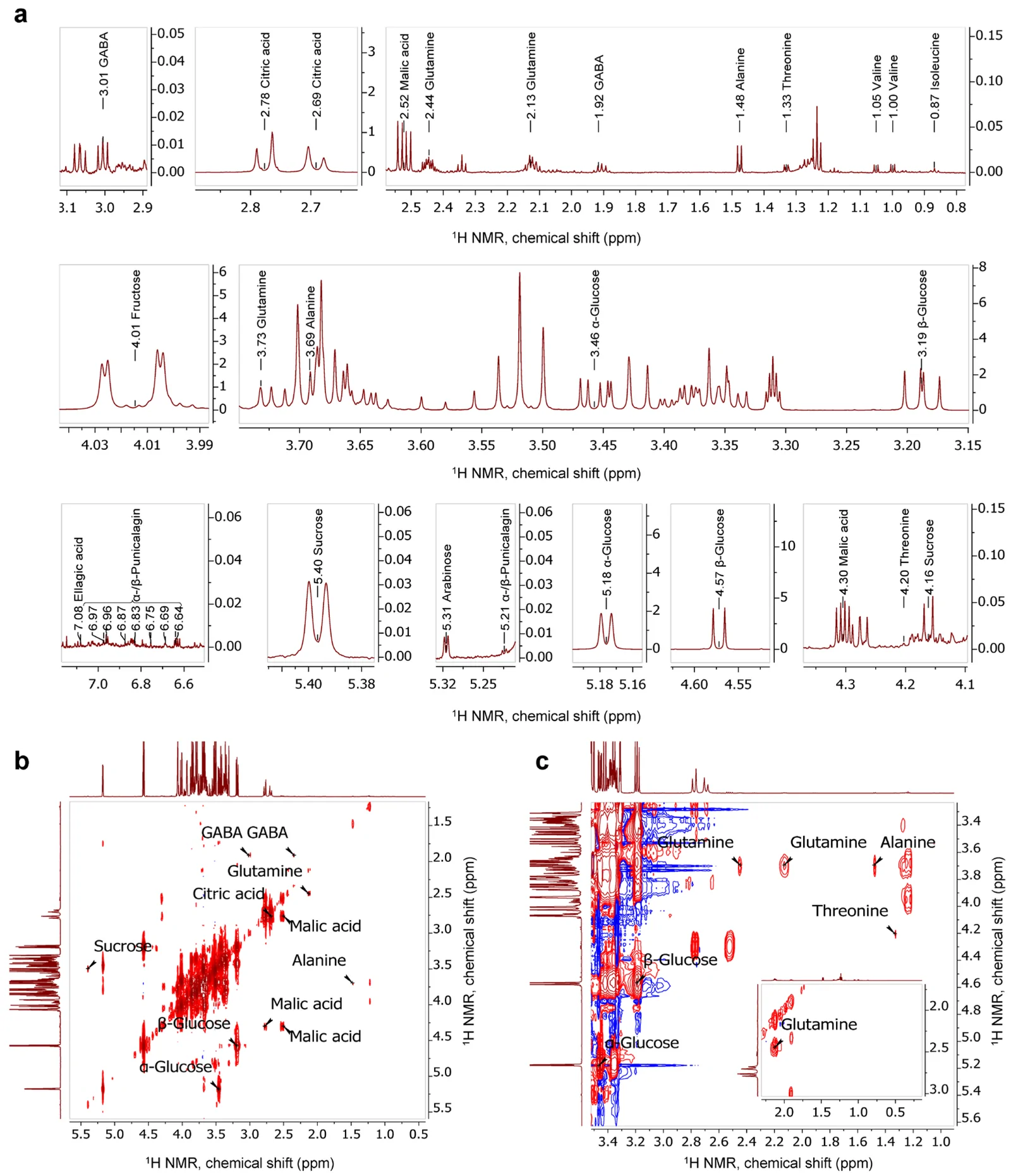
Metabolite profiling of pomegranate juice using NMR spectroscopy. Representative proton (**a**) and two-dimensional COSY (**b**) and TOCSY (**c**) spectra with assigned signals of the identified metabolites.

The proton NMR spectrum is a valuable source of information for the main primary metabolites and some highly abundant secondary metabolites. Within the PGJ, some common sugars, amino and organic acids were identified after comparison with spectral data from the literature [18,19,20,21,22,23] or exploiting available spectral data from the Biological Magnetic Resonance Data Bank [24] and Human Metabolome Database [25]. Representative spectra are provided in Figure 1.

The sugar region of the proton NMR spectra (3–5 ppm) revealed signals corresponding to *α*-glucose at 5.18 and 3.46 ppm, *β*-glucose at 4.57 and 3.19 ppm, fructose at 4.02 ppm, sucrose at 4.16 and 5.40 ppm and arabinose at 5.31 ppm (Figure 1a). Within the aliphatic region (0–4.5 ppm), the signals corresponding to several amino acids were detected: alanine at 1.48 and 3.69 ppm, isoleucine at 0.87 ppm, valine at 1.00 and 1.05 ppm, threonine at 1.33 and 4.2 ppm, glutamine at 2.13, 2.44 and 3.73 ppm, and γ-amino-butyrate (GABA) at 1.92, 2.34 and 3.01 ppm (Figure 1a).

Within the PGJ spectra, the characteristic resonances for citric acid at 2.69 and 2.78 ppm, as well as malic acid at 2.52, 2.77, and 4.30 ppm, are readily observed. In addition to the primary metabolites identified, signals corresponding to ellagic acid and α-/β-punicalagin signals at 7.08, 6.97, 6.96, 6.87, 6.83, 6.75, 6.69, and 6.64 ppm were assigned within the aromatic region of the proton spectrum (Figure 1a).

Chemical shifts (δ) and the corresponding coupling constants (*J*) identified by their relevant ^1^H NMR spectra are listed in Supplementary Table S1. In addition to the proton NMR, the respective ^1^H-^1^H cross-peaks for *α*-glucose, *β*-glucose, alanine, glutamine, GABA, citric acid (CA) and malic acid (MA) were found within the two-dimensional homonuclear correlation spectroscopy (COSY, Figure 1b) and total correlation spectroscopy (TOCSY) spectra (Figure 1c). Chemical shifts (*δ*) of the protons from the assigned cross-peaks are listed in Supplementary Table S1.

### 2.2. Quantification of Punicalagin in Pomegranate Juice Using UPLC-qToF-MS

**Figure 2:**
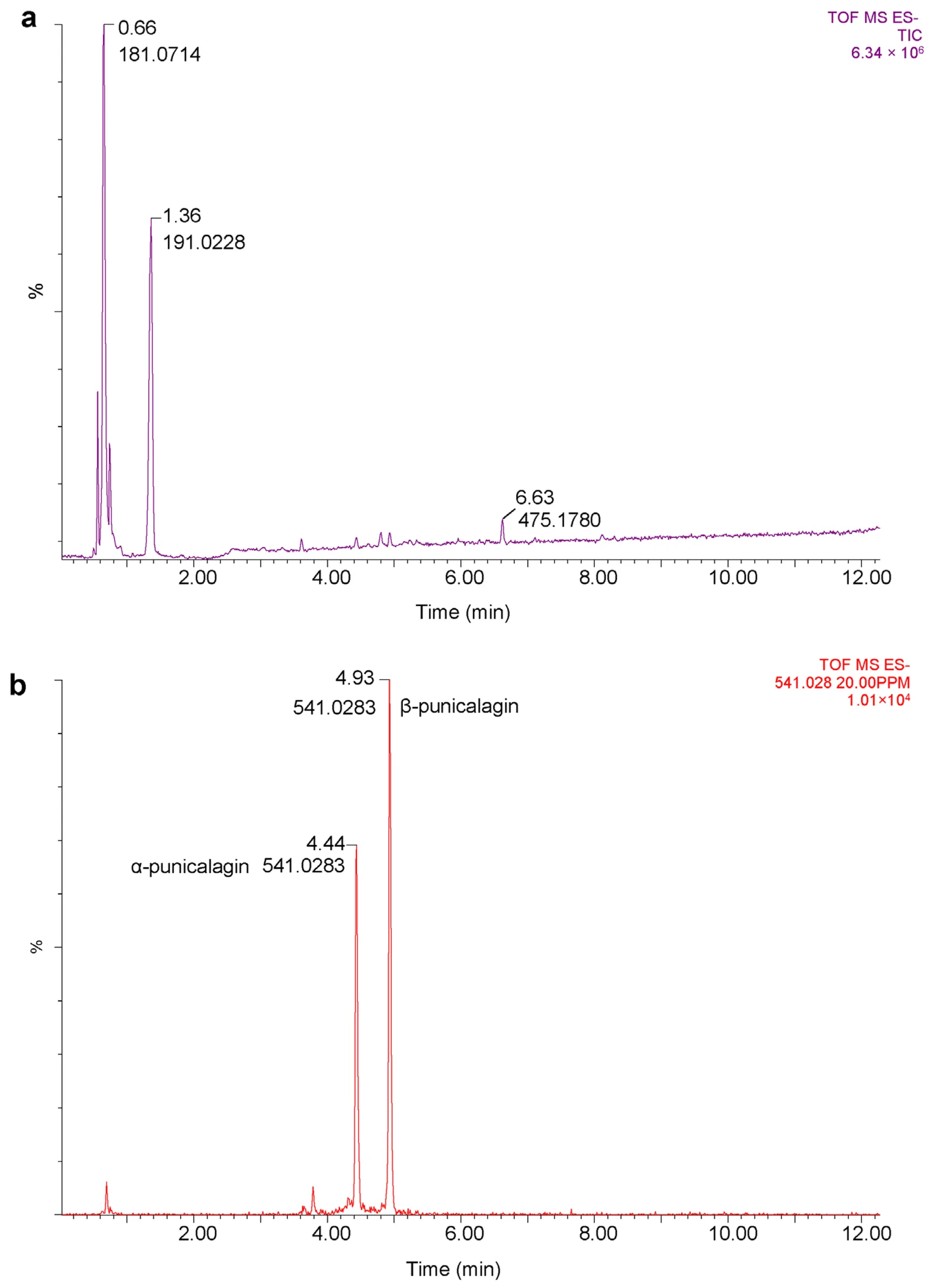
Representative UPLC-qToF-MS chromatograms of pomegranate juice. (**a**) Total ion chromatogram (TIC) of PGJ 1 mg/mL. (**b**) Extracted ion chromatogram (EIC) for α-/β-punicalagin–[M-2H]^2−^ *m*/*z* = 541.0283 in PGJ.

Punicalagin was identified in lyophilized pomegranate juice samples by UPLC-qToF-MS based on retention time matching with a reference standard and accurate mass measurement. The compound occurs as two stereoisomers, differing in the orientation of the hydroxyl group at the anomeric carbon of the glucosyl moiety [26]. The α- and β-isomers of punicalagin eluted at retention time 4.44 and 4.94 min, respectively, allowing us to distinguish the two isomers (Figure 2). This identification was based on previous studies of their elution profile [21].

Punicalagin was detected in negative ionisation mode as a deprotonated molecular ion [M-H]^−^ *m*/*z* 1083.0579 and a double-charged molecular ion [M-2H]^2−^ at 541.0283. The doubly charged ion was selected for quantification due to its higher signal intensity and improved peak shape.

Quantification was carried out using an external calibration curve prepared from punicalagin standard solutions in the concentration range of 1.5625–100 μg/mL. The calibration curves were plotted for each isomer. The calibration curves exhibited good linearity, with a correlation coefficient (R^2^) of 0.9999 (α-punicalagin) and 0.9998 (β-punicalagin). The limits of detection (LOD) and quantification (LOQ) were 0.43 and 1.30 μg/mL, respectively. Punicalagin was quantified in the analysed pomegranate juice samples at a concentration of 2.4 (0.127) μg/mg lyophilized pomegranate juice. Data are expressed as mean (SD) of three independently prepared sample solutions from the same PGJ preparation, each analysed in triplicate.

### 2.3. Both PGJ and PUN Enhance Stress Resilience in C. elegans

As a preliminary assessment of tolerability, we evaluated the effects of PGJ and PUN across a range of concentrations. No statistically significant changes in *C. elegans* viability were observed following 48 h exposure to PGJ (10, 50, 100, and 200 μg/mL) or PUN (25, 50, 100, and 250 μM) at the tested concentrations (Supplementary Figure S1a,b). Based on these results, PGJ (50, 100, and 200 μg/mL) and PUN (25, 50, and 100 μM) were selected for subsequent experiments.

We next assessed whether PGJ modulates healthspan-related parameters, including lifespan, age-associated locomotor decline, and fertility. The PGJ treatment significantly attenuated age-related locomotor decline, increasing mean body bends per 30 s by 8% on day 5 and 14% on day 10, compared with the vehicle-treated group (Supplementary Figure S1d). PGJ at 200 μg/mL also significantly increased lifespan, whereas no significant effects on fertility were observed (Supplementary Figure S1c,e).

**Figure 3:**
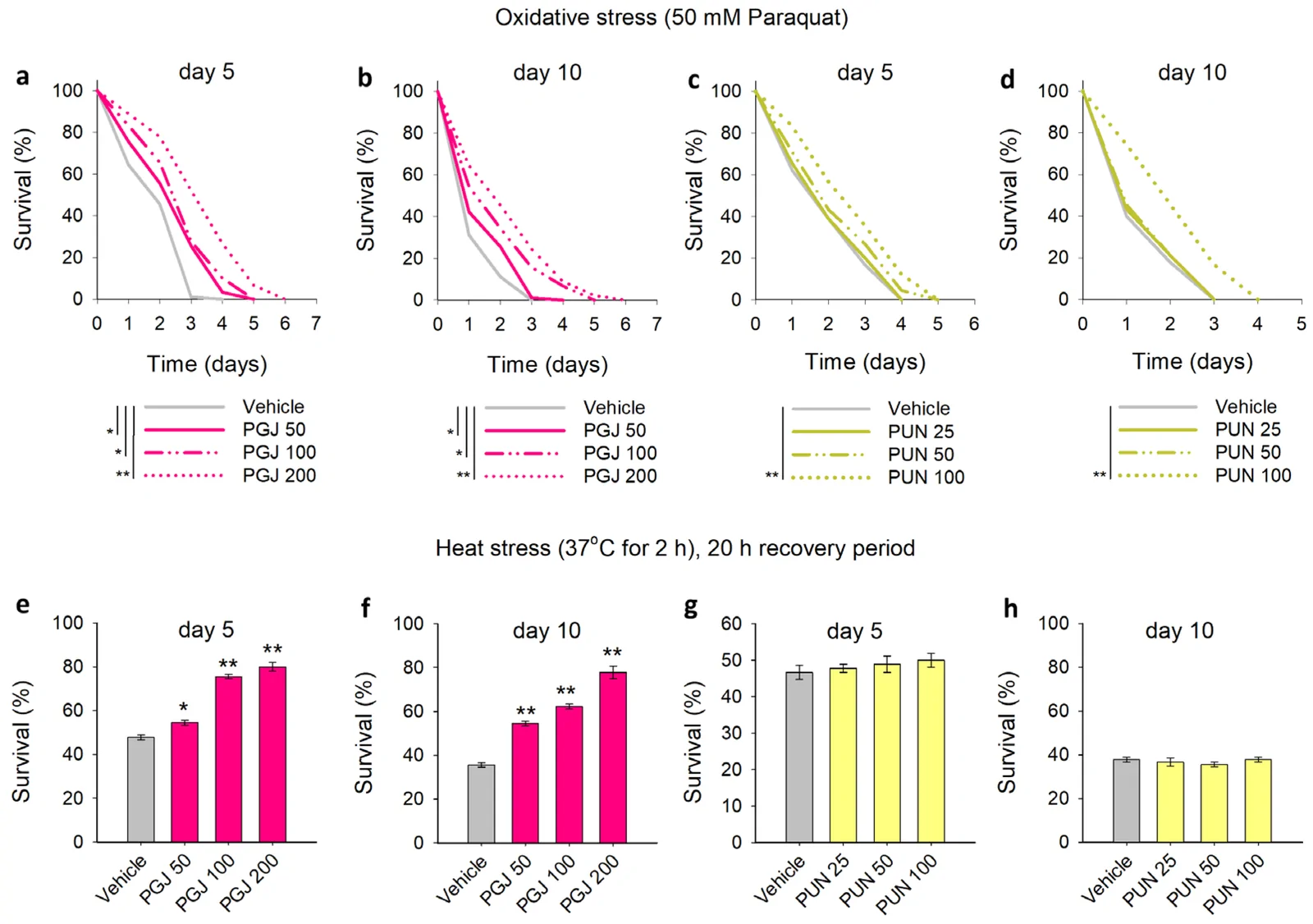
Differential effects of pomegranate juice and punicalagin on oxidative and heat stress resilience. Percentage survival of *C. elegans* subjected to (**a**–**d**) oxidative stress (50 mM paraquat measured at 24 h intervals) and (**e**–**h**) heat stress (37 °C for 2 h) followed by a 20 h recovery period under normal conditions of N2 worms and treated with PGJ (50, 100, and 200 μg/mL, **a**,**b**,**e**,**f**) and PUN (25, 50 and 100 μM, **c**,**d**,**g**,**h**) on two time points–day 5 (**a**,**c**,**e**,**g**) and day 10 (**b**,**d**,**f**,**h**). Data from oxidative stress survival are plotted as survival curves, sample size: n > 90 per treatment. Data were represented using Kaplan–Meier analysis, and *p* values were calculated using the log-rank test. Survival after acute heat stress exposure (day 5 and day 10 of lifespan). The results are represented as percentage survival as mean ± SEM, n = 90, * *p* < 0.05, ** *p* < 0.01 (one-way ANOVA, followed by Tukey post hoc test).

Stress resilience is an important component of organismal health and is closely linked to metabolic adaptation during ageing. We therefore examined whether PGJ (50, 100, 200 μg/mL) and PUN (25, 50, 100 μM) enhance resistance to thermal and oxidative stress. The results demonstrated that PGJ increased resistance to both stressors in a concentration-dependent manner at both ages examined. The strongest effect was observed at 200 μg/mL, which increased mean survival by up to 1.67-fold under oxidative stress (Figure 3a) and heat stress (Figure 3e) on day 5. On day 10 of adulthood, PGJ at the same concentration increased mean survival by 1.73-fold under oxidative stress (Figure 3b) and 2.25-fold under heat stress (Figure 3f) relative to the vehicle-treated group. In contrast, PUN enhanced resistance to oxidative stress only at 100 μM, increasing mean survival by 1.32-fold on day 5 (Figure 3c) and 1.50-fold on day 10 (Figure 3d) relative to the vehicle-treated group. Thus, PGJ produced a broader stress-resilience phenotype, whereas the effect of PUN was restricted to oxidative stress under the conditions tested.

### 2.4. Pomegranate Juice and Punicalagin Influence Metabolic Plasticity and Mitochondrial Function, Correlating with Changes in Body Morphology

Metabolic dysfunction is frequently accompanied by altered lipid homeostasis and mitochondrial impairment [27]. We therefore examined whether PGJ and PUN alter neutral lipid-associated fluorescence and mitochondrial function under basal conditions [27,28].

**Figure 4:**
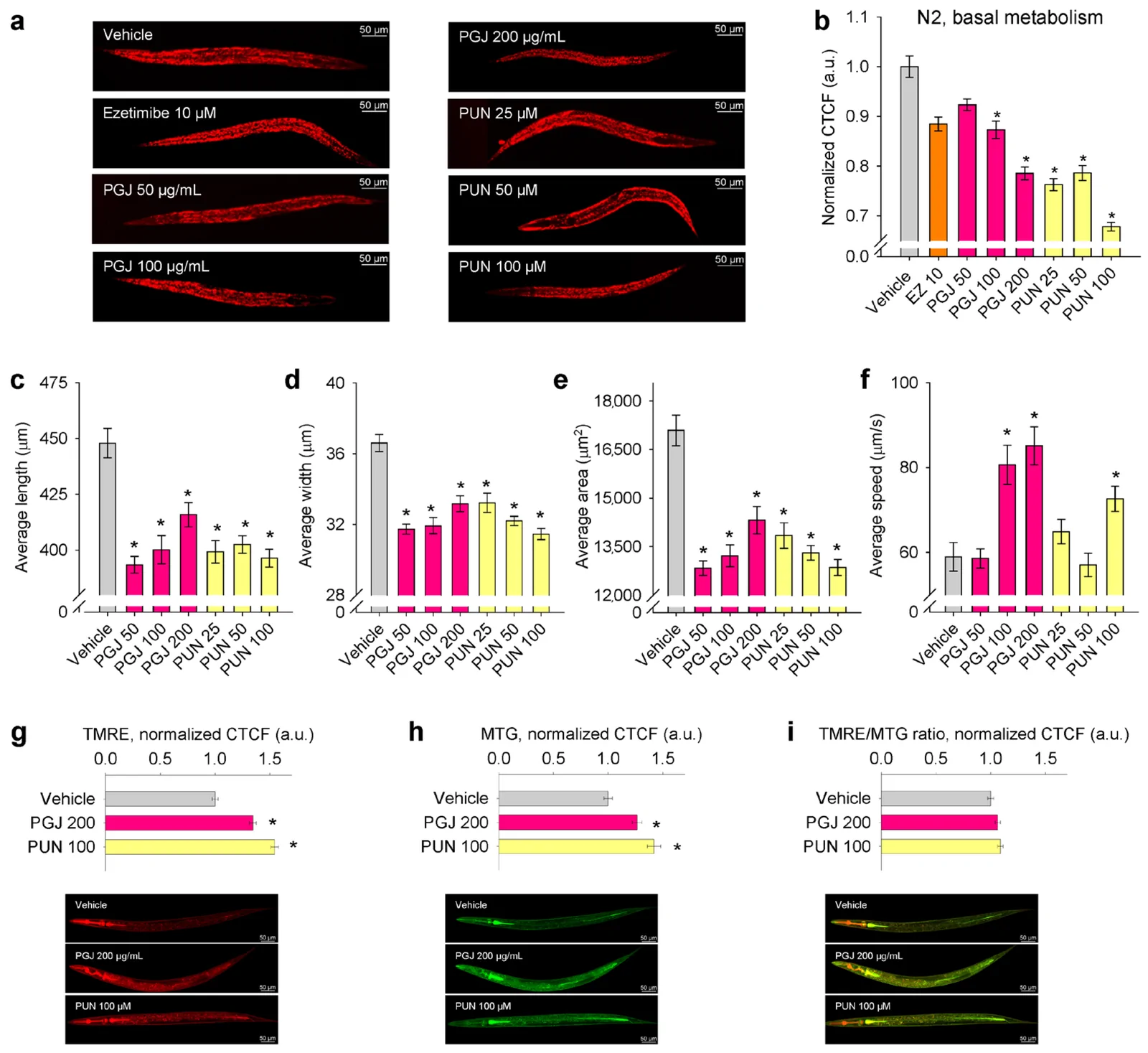
Both PGJ and PUN improve basal metabolism and mitochondrial homeostasis. (**a**) Representative confocal photographs at 20× magnification (scale bar of 50 μm) of Nile red staining of lipid deposition in wild-type N2 worms (late L4 larval stage) with PGJ (50, 100, and 200 μg/mL) and PUN (25, 50 and 100 μM), ezetimibe (EZ, 10 μM) or vehicle (0.4% DMSO). (**b**) Quantification of lipid accumulation as normalised to vehicle group corrected total cell fluorescence (CTCF), represented as arbitrary units (a.u.). (**c**) Mean length, (**d**) width, (**e**) area, and (**f**) average worm speed were measured and analysed using the WormLab software (version 2023.1.1; n = 110–130). (**g**) Representative confocal images (20× magnification, scale bar: 50 μm) showing TMRE fluorescence (red channel) and quantification of mitochondrial membrane potential-dependent fluorescence. Red channel fluorescence intensity was calculated for each worm as Corrected Total Cell Fluorescence (CTCF): CTCF = Integrated Density − (Area × Mean background fluorescence). Values were then normalised to the mean CTCF of the vehicle (-G)-treated group. Data are presented as mean ± SEM (n = 60 worms from three independent biological replicates). (**h**) Representative confocal images (20× magnification, scale bar: 50 μm) showing green channel fluorescence (MTG) and quantification of mitochondrial mass-dependent fluorescent signal. Green channel fluorescence intensity was calculated as CTCF and normalised to vehicle (-G) as in (**g**). n = 60, 3 replicates. Data for (**a**–**i**) are presented as mean ± SEM, significance between the groups was assigned as * *p* < 0.05, vs. vehicle (ANOVA on ranks with Dunn’s post hoc test). For basal Nile Red-associated fluorescence (**b**), the overall treatment effect was significant (Kruskal–Wallis H(7) = 233.37, *p* < 0.001; ε^2^ = 0.286).

To assess the impact of PGJ and PUN on basal lipid status, neutral lipid accumulation was quantified using Nile red staining. Ezetimibe, a clinically used inhibitor of intestinal cholesterol absorption [29,30], was included as a pharmacological reference compound. Data revealed that both treatments significantly modulated Nile red-derived fluorescence (Figure 4a,b), with PGJ (200 µg/mL) and PUN (100 μM) producing the strongest reductions, reaching 0.785 (95% CI, 0.760–0.811) and 0.678 (95% CI, 0.661–0.695), respectively. In contrast, EZ did not significantly alter the basal Nile red-derived fluorescence signal (Figure 4a,b).

Automated image-based analysis further revealed significant reductions in body length (Figure 4c), width (Figure 4d), and area (Figure 4e), accompanied by increased average worm speed (Figure 4f). As the strongest phenotypic effects were observed at the highest concentrations tested, PGJ (200 μg/mL) and PUN (100 μM) were selected for subsequent analyses of mitochondrial function.

Mitochondrial staining revealed a statistically significant increase in mitochondrial membrane potential-dependent fluorescent signal (Figure 4g), reaching up to 1.33-fold and 1.49-fold following treatment with 200 µg/mL PGJ and 100 µM PUN, respectively. Mitochondrial mass-dependent fluorescence was also increased by 1.24-fold and 1.34-fold following PGJ and PUN treatment, respectively (Figure 4h). However, the ratio between the fluorescence dependent on membrane potential and the fluorescence dependent on mitochondrial mass, which is considered an indicator of membrane potential changes, remained unchanged (Figure 4i).

### 2.5. Lipid-Associated Responses to PGJ and PUN in an NHR-49/MDT-15 Gain-of-Function Mutant Under Glucose-Enriched Conditions

Given the observed effects of PGJ and PUN on lipid homeostasis, we next examined whether these effects are maintained under conditions of excessive glucose supplementation, a commonly used *C. elegans* model for assessing the metabolic regulatory potential of natural compounds [31,32].

**Figure 5:**
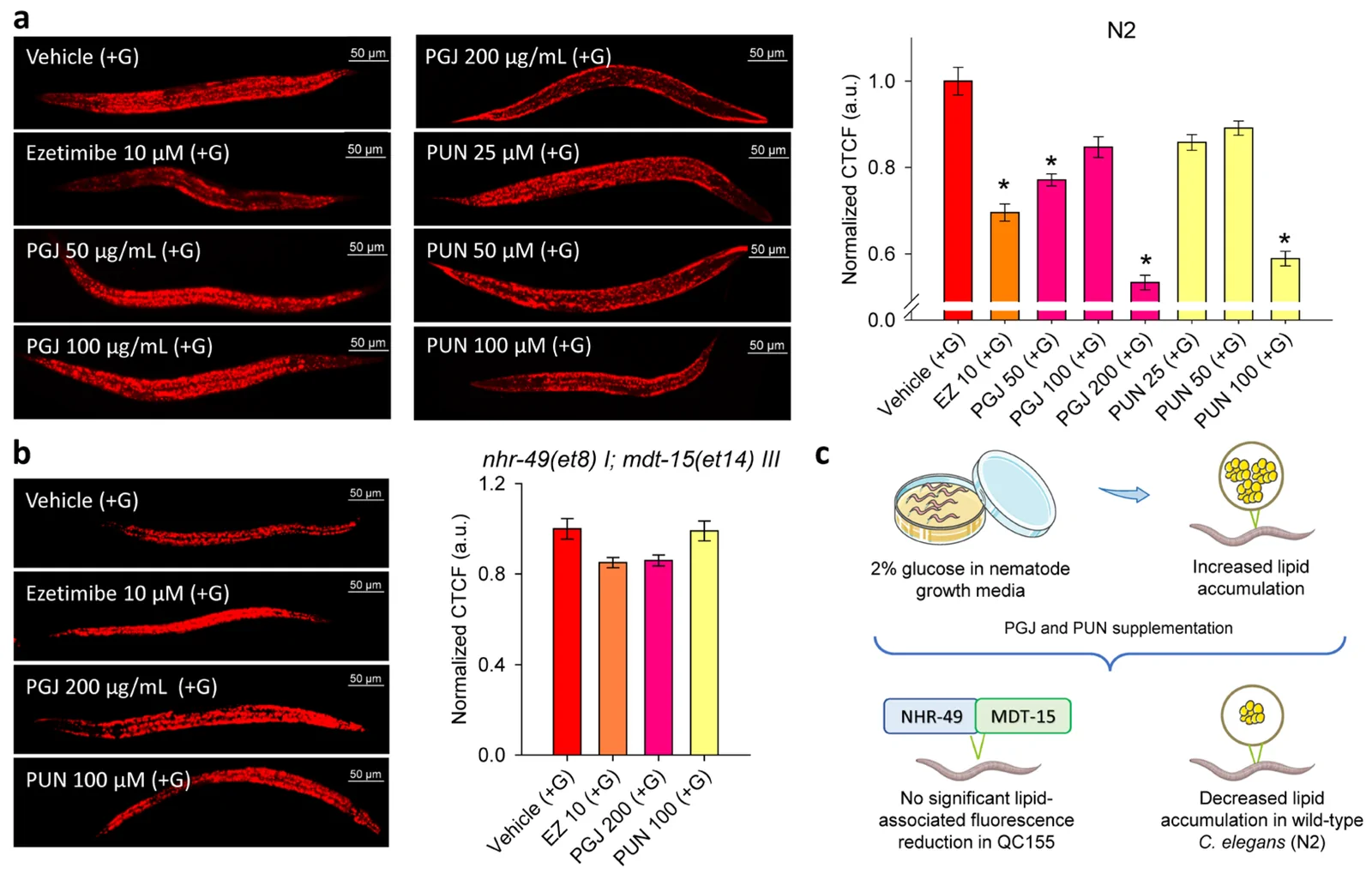
Lipid-reducing effects of PGJ and PUN under glucose-enriched conditions suggest modulation through NHR-49 and MDT-15. Representative confocal images of Nile red lipid staining of glucose-supplemented worms (late L4 larval stage) treated for 24 h with *P. granatum* juice (PGJ, 50, 100, and 200 μg/mL), punicalagin (PUN, 25, 50, and 100 μM), ezetimibe (EZ, 10 μM), or vehicle (0.4% DMSO) and quantification of lipid accumulation presented as normalised corrected total cell fluorescence (CTCF) expressed in arbitrary units (a.u.) in (**a**) N2 (wild type) nematodes and (**b**) QC155 *[nhr-49(et8) I; mdt-15(et14) III]* mutant strain. Values are presented as mean ± SEM; * *p* < 0.05 compared to vehicle (+G) group, n = 60, 3 replicates, ANOVA on ranks. (**c**) Graphical summary of the glucose-induced metabolic challenge and the effects of PGJ and PUN. For glucose-enriched Nile red-associated fluorescence (**a**), H(7) = 269.85, *p* < 0.001, with an effect size of ε^2^ = 0.369.

Under glucose-enriched conditions, PGJ (200 μg/mL) and PUN (100 μM) reduced normalised Nile red-associated fluorescence to 0.533 (95% CI, 0.500–0.567) and 0.589 (95% CI, 0.556–0.623), respectively, whereas EZ (10 μM) reduced the signal to 0.696 (95% CI, 0.656–0.736) (Figure 5a).

We next investigated whether the NHR-49/MDT-15 regulatory axis contributes to the reduction in glucose-induced neutral lipid accumulation associated with PGJ and PUN treatment. For this purpose, we used the QC155 mutant strain, carrying gain-of-function alleles in both *nhr-49* and *mdt-15*, and assessed lipid accumulation under glucose-enriched conditions. In contrast to wild-type animals, PGJ (200 μg/mL), PUN (100 μM), and EZ (10 μM) did not significantly reduce neutral lipid-associated fluorescence in QC155 animals relative to the corresponding vehicle (Figure 5b). These findings suggest that the lipid-associated responses to PGJ and PUN may be influenced by the altered NHR-49/MDT-15 genetic background under glucose-induced metabolic stress (Figure 5c).

### 2.6. The PGJ and PUN Regulate Genes Involved in Lipid and Energy Metabolism

**Figure 6:**
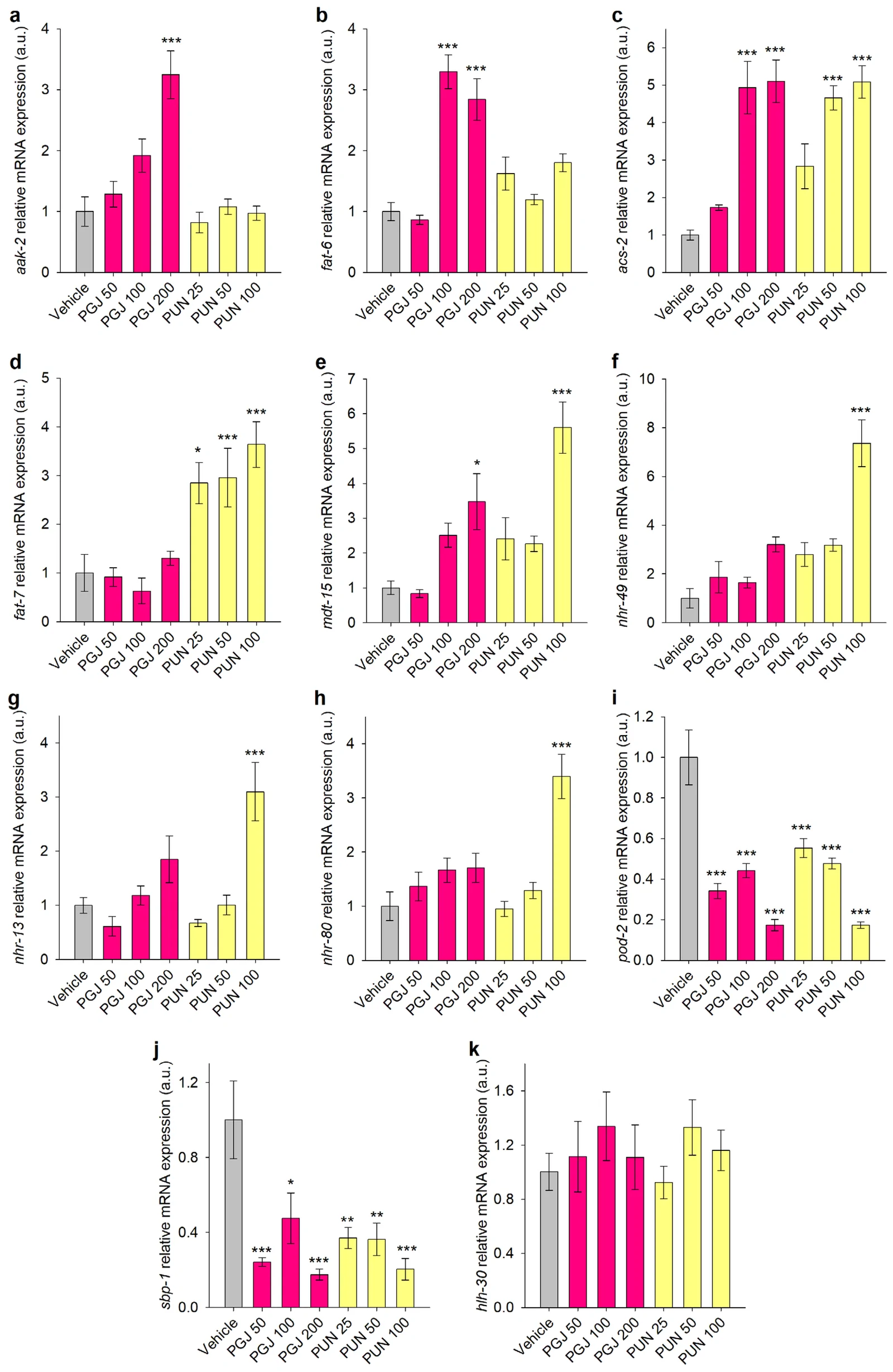
Gene expression profiles of metabolic and lipid-related genes following PGJ or PUN treatment in *C. elegans*. Normalised relative mRNA expression levels in arbitrary units (a.u.) of genes associated with metabolism regulation (**a**) *aak-2*, (**b**) *fat-6*, (**c**) *acs-2*, (**d**) *fat-7*, (**e**) *mdt-15*, (**f**) *nhr-49*, (**g**) *nhr-13*, (**h**) *nhr-80*, (**i**) *pod-2*, (**j**) *sbp-1*, and (**k**) *hlh-30* following treatment with vehicle, PGJ, or PUN. The genes *iscu-1* and *pmp-3* were used as reference genes to normalise target gene expression. Data are expressed as mean ± SEM from three independent biological experiments, each measured in technical triplicate. * *p* < 0.05, ** *p* < 0.01, and *** *p* < 0.001 compared to the vehicle (one-way ANOVA). Effect sizes for the overall treatment effect were: *aak-2*, η^2^ = 0.588; *fat-6*, η^2^ = 0.670; *acs-2*, η^2^ = 0.606; *fat-7*, η^2^ = 0.515; *mdt-15*, η^2^ = 0.524; *nhr-49*, η^2^ = 0.629; *nhr-13*, η^2^ = 0.489; *nhr-80*, η^2^ = 0.527; *pod-2*, η^2^ = 0.707; *sbp-1*, η^2^ = 0.440; and, *hlh-30*, η^2^ = 0.059.

We next sought to explore the molecular basis of the metabolic effects of PGJ (50, 100, 200 μg/mL) or PUN (25, 50, 100 μM) by analysing the expression of key genes involved in nutrient sensing, lipid turnover, and metabolic regulation. Our results showed that PGJ at 200 µg/mL upregulated the 5′-AMP-activated protein kinase catalytic subunit alpha-2 (*aak-2*), whereas PUN did not alter its expression (Figure 6a).

Similarly, only PGJ (100 and 200 µg/mL) increased the expression of the Δ9-fatty acid desaturase *fat-6* (Figure 6b), while PUN again showed no effect. Interestingly, *acs-2*, a member of the fatty acid CoA synthetase family involved in β-oxidation, was upregulated by both PGJ (100, 200 μg/mL) and PUN (50, 100 μM) increasing the relative gene expression to 4.93- and 5.10-fold for PGJ and 4.66- and 5.08-fold for PUN, respectively (Figure 6c). Conversely, *fat-7* expression was increased in a concentration-dependent manner only following PUN treatment (25, 50, and 100 μM), reaching 2.85-, 2.96-, and 3.64-fold, respectively (Figure 6d).

We also examined the basal expression of *mdt-15* and *nhr-49*, given the altered lipid-associated responses observed in the QC155 mutant background under glucose-enriched conditions. The data revealed that mdt-15 (Figure 6e) was upregulated by both 200 µg/mL PGJ (3.47) and 100 µM PUN (5.6), whereas *nhr-49* expression was markedly elevated up to 7.36-fold only following PUN treatment (100 µM; Figure 6f). We further examined two additional nuclear hormone receptors, *nhr-13* and *nhr-80*. Both showed a similar response pattern, with significant upregulation observed only following treatment with PUN at 100 μM (Figure 6g,h).

Furthermore, both PGJ and PUN reduced the expression of *pod-2* (Figure 6i), encoding acetyl-CoA carboxylase, with the lowest expression levels reaching 0.03 following PGJ treatment at 100 μg/mL and 0.02 following PUN treatment at 100 μM. Similarly, *sbp-1*, the *C. elegans* homologue of sterol regulatory element-binding protein 1 and a key regulator of fatty acid synthesis, was downregulated to 0.17 and 0.20 following PGJ (100 μg/mL) and PUN (100 μM) treatment, respectively (Figure 6j). No significant changes were observed in the expression of *hlh-30* (Figure 6k), a transcriptional regulator of autophagy.

### 2.7. The PGJ and PUN Engage Partially Overlapping but Distinct Metabolic Response Profiles

**Figure 7:**
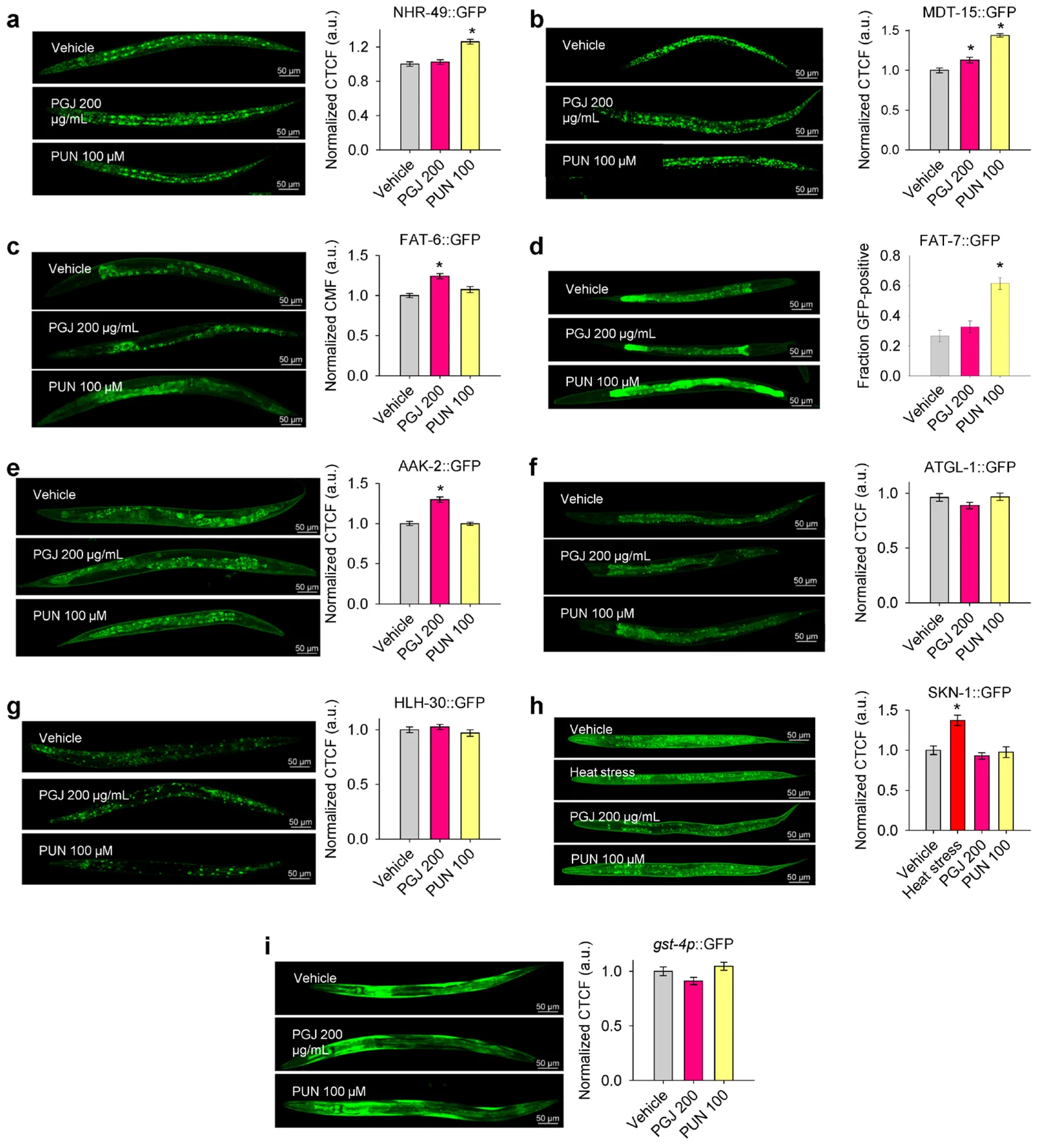
Treatment with PGJ and PUN regulates overlapping metabolic pathways, with MDT-15 as a shared regulatory node. Representative fluorescent confocal microphotographs at 20× magnification (scale bar of 50 μm) of *C. elegans* strains treated with vehicle, PGJ (200 μg/mL) or PUN (100 μM), as follows: (**a**) PHX3258 *nhr-49(syb3258[NHR-49::GFP])* I, (**b**) IJ1651 *mdt-15(yh44[mdt-15::degron::EmGFP])* III, (**c**) PHX649 *fat-6(syb649[FAT-6::GFP])* IV, (**d**) DMS303 *nIs590 [pfat-7FAT-7::GFP + lin-15(+)]* V, (**e**) AGD467 *uthEx490 [aak-2 (intron 1)::AAK-2(aa1–aa321 T172D)::GFP + pcrtc-1CRTC-1::tdTomato + rol-6(su1006)]*, (**f**) VS20 *hjIs67 [patgl-1ATGL-1::GFP + MEC-7::RFP]*, (**g**) MAH240 *sqIs17 [phlh-30HLH-30::GFP + rol-6(su1006)]*, (**h**) LD1 *ldIs7 [SKN-1B/C::GFP + rol-6(su1006)]*, and (**i**) CL2166 *dvIs19 [(pAF15)pgst-4GFP::NLS]* III. Quantification of fluorescence intensity for (**a**,**b**,**e**–**i**) is presented as corrected total cell fluorescence (CTCF), and for (**c**) as corrected mean fluorescence (CMF). All values are normalised to the respective vehicle group and shown in arbitrary units (a.u.). Data are presented as means ± SEM, n = 60, * *p* < 0.05, compared to the vehicle (ANOVA on ranks). For (**d**), data are presented as the fraction of GFP-positive animals ± standard error of proportion (SEP), (n > 120), compared to the vehicle (*t*-test).

To further characterise the molecular responses associated with PGJ and PUN supplementation, we analysed a panel of transgenic reporter strains expressing GFP- or tdTomato-tagged translational fusion proteins corresponding to the selected molecular targets of interest. The obtained data revealed that NHR-49 protein abundance was increased to 1.26 only following treatment with 100 μM PUN (Figure 7a).

Analysis of the GFP-tagged MDT-15 reporter further supported its involvement in the response to both PGJ (200 μg/mL) and PUN (100 μM), with protein abundance increasing to 1.13- and 1.44-fold, respectively (Figure 7b). Among the fatty acid desaturases, FAT-6 protein levels increased to 1.24-fold following PGJ treatment (200 μg/mL), whereas the proportion of FAT-7::GFP-positive animals increased significantly only following PUN treatment (100 μM), as reflected by an increase in the proportion of GFP-positive animals from 0.27 to 0.62 (Figure 7c,d). Similarly, AAK-2::GFP fluorescence increased 1.30-fold exclusively following PGJ treatment (200 μg/mL) (Figure 7e).

The ATGL-1 was included as a marker of lipid catabolism; however, neither PGJ nor PUN significantly altered ATGL-1 protein abundance (Figure 7f). Similarly, HLH-30 protein levels remained unchanged following either treatment (Figure 7i). No significant changes were observed in the SKN-1 reporter or its downstream target gst-4 (Figure 7g,h), indicating that these treatments did not markedly alter these stress-response readouts under the conditions tested. Together, these findings indicate partially overlapping but distinct molecular responses to PGJ and PUN, with MDT-15 representing a shared treatment-responsive component (Figure 7b).

### 2.8. Treatment with PUN Improves Locomotor Performance Under Conditions of Aβ-Induced Proteotoxicity

**Figure 8:**
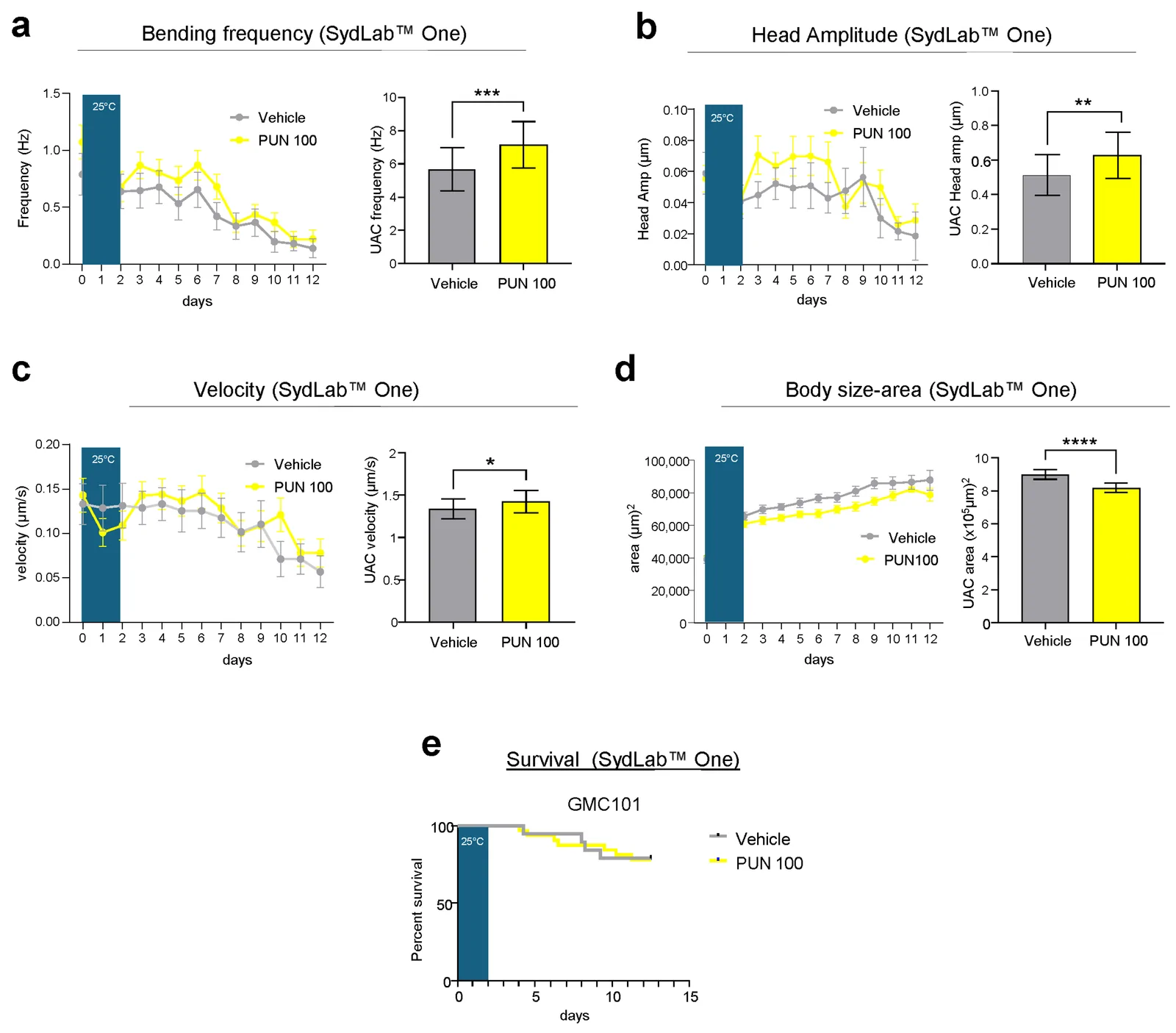
SydLab™ One analyses for phenotypic, healthspan, and survival parameters of PUN-treated nematodes under Aβ-induced proteotoxicity. L4-staged nematodes of the GMC101 strain were loaded and maintained in the SydLab One platform for 15 days upon treatment with DMSO 0.4% (Vehicle) or PUN 100 μM. Aβ expression was induced by incubating animals at 25 °C for 48 h (from L4 to Day2 of adulthood). (**a**–**d**) Daily measurements (**left** panels) and total values across the entire experimental duration (**right** panels) are shown for bending frequency (**a**), head amplitude (**b**), velocity (**c**) and body size (**d**). Data are presented as mean ± SEM, * *p* < 0.05, ** *p* < 0.01, *** *p* < 0.001, **** *p* < 0.0001 compared to the vehicle (Student’s *t*-test). (**e**) Kaplan–Meier survival curves showing the percentage of alive nematodes over the 15-day experimental period.

Having established PUN-associated metabolic and regulatory responses, we next asked whether PUN was also associated with preserved function under chronic proteotoxic challenge. We employed the well-established Alzheimer’s disease *C. elegans* model GMC101, which overexpresses the pathogenic amyloid-β peptide Aβ_1–42_ in body wall muscle cells (Figure 8a–c).

In this strain, Aβ expression and aggregation remain relatively low at the permissive temperature of 20 °C, whereas a temperature shift to 25 °C strongly induces Aβ accumulation, leading to progressive locomotor impairment [33,34]. To obtain an unbiased quantitative assessment of health-related phenotypes, control and PUN-treated nematodes of the GMC101 strain were analysed using the fully automated SydLab™ One platform (Nagi Bioscience). Continuous high-content imaging enabled the longitudinal evaluation of motility, worm metric parameters and survival rate, following Aβ induction. The analysis of motility parameters revealed a substantial improvement in locomotor behaviour upon PUN treatment. Specifically, bending frequency, head amplitude and average velocity were significantly elevated in PUN-treated nematodes (Figure 8a–c), indicating an overall enhancement of locomotor capacity.

Notably, these improvements were consistent with the effects observed in the N2 wild-type background (Figure 4f and Supplementary Figure S1). In parallel, PUN-treated GMC101 animals displayed a reduction in body size metrics compared to the vehicle-treated controls (Figure 8d), which is coherent with the results from body parameters of wild-type nematodes (Figure 4c–e). In contrast to the marked improvements observed in the locomotor behaviour and worm metric parameters, PUN treatment did not significantly affect survival, which was monitored up to day 15 of the assay.

Collectively, these findings show that PUN improved several locomotor parameters in the GMC101 animals under Aβ-induced proteotoxic stress. However, because parallel experiments were not performed in wild-type animals under the same temperature-shift protocol, the present data do not distinguish between disease-specific effects and a more general enhancement of locomotor performance.

### 2.9. Supplementation with PGJ and PUN Modulates Shared Molecular Pathways for Improving Metabolic Homeostasis and Stress Resilience

**Figure 9:**
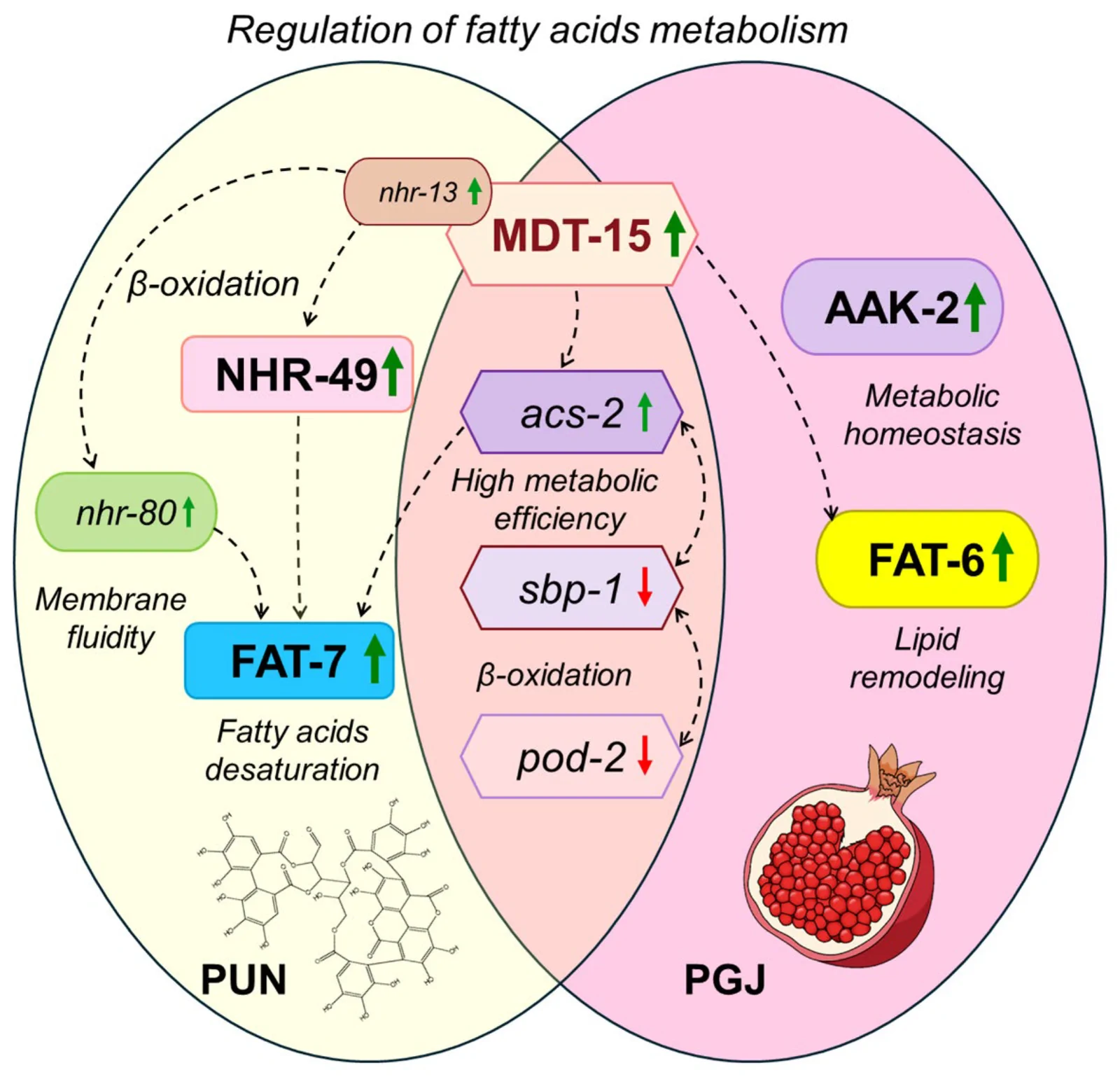
Schematic summary of shared and distinct molecular responses to pomegranate juice (PGJ) and punicalagin (PUN) in *C. elegans.* Upward and downward arrows indicate treatment-associated increases and decreases, respectively. The dashed arrows indicate possible molecular interactions. The proposed model illustrates potential molecular interactions and their metabolic implications rather than established causal interactions.

To integrate the observed molecular responses, a schematic model summarising the shared and treatment-specific changes associated with PGJ and PUN supplementation is presented in Figure 9. Both treatments increased MDT-15-associated responses and *acs-2* expression while downregulating *sbp-1* and *pod-2*. PGJ preferentially increased AAK-2 and FAT-6 responses, whereas PUN was associated with stimulated NHR-49 and FAT-7 reporter signals, and upregulated *nhr-13* and *nhr-80*. These findings indicate partially overlapping but distinct molecular profiles associated with lipid homeostasis under the investigated conditions.

## 3. Discussion

Due to its rich content of bioactive phytochemicals, pomegranate juice exhibits a broad spectrum of biological activities, including cardiometabolic, anti-inflammatory and neuroprotective effects [14,35,36]. These properties have supported its long-standing use as a medicinal food and have contributed to increasing interest in its potential role in the prevention and management of chronic metabolic disorders [5,13,37]. Among its major phytochemicals, ellagitannins, particularly punicalagin, are considered important contributors to the health-promoting properties of pomegranate juice [36]. Consistent with these observations, clinical studies have reported that long-term pomegranate juice consumption improves several cardiovascular and metabolic risk markers, including blood pressure, LDL oxidation, carotid intima-media thickness and postprandial glycaemic control [14,35,38]. Together, these findings support the health-promoting potential of pomegranate juice while highlighting the need to better define the molecular mechanisms underlying its biological effects.

This study shows that PGJ and PUN are associated with partially overlapping but distinct metabolic responses in *C. elegans*, together with improvements in stress resilience and locomotor parameters. The work integrates chemical characterisation of the juice matrix with bioactivity mechanistic studies. The principal observation is that both treatments elicited lipid-associated responses accompanied by changes in NHR-49/MDT-15-related molecular readouts, although their downstream molecular signatures differed.

Metabolite profiling of *P. granatum* products, including extracts and juices, using chromatographic techniques [21,39,40] and NMR spectroscopy [41,42,43] has reported phenolic compounds as the predominant secondary metabolites. Among these, ellagitannins represent an important class of phytochemicals, with punicalagin being one of the most abundant constituents of pomegranate juice [44]. Although PUN has been extensively quantified across different pomegranate products, particularly high levels have also been reported in fruit peels [39,45,46,47]. In the present study, NMR profiling of PGJ revealed the presence of sugars, amino and organic acids, as well as the polyphenols ellagic acid and punicalagin. Given the lower sensitivity of the NMR technique, UPLC-qToF-MS was subsequently used for quantification of PUN, yielding a content of 2.4 μg/mg PGJ. Although glucose and fructose were identified in PGJ by NMR spectroscopy, their individual concentrations were not quantified, and a sugar-matched control was not included. Given the relatively low concentration of lyophilized PGJ used (up to 200 μg/mL), the contribution of juice-derived sugars to the overall sugar load under glucose-enriched conditions is expected to be limited. Nevertheless, their potential influence on the observed metabolic responses cannot be entirely excluded.

In the present study, PUN was selected for independent biological evaluation as a chemically identified constituent of the investigated PGJ preparation, rather than to reproduce its concentration within the juice or to determine its quantitative contribution to the overall biological activity of PGJ. Based on the quantified PUN content of PGJ, treatment with 200 μg/mL PGJ corresponds to approximately 0.44 μM PUN, whereas PUN was tested at concentrations up to 100 μM. Therefore, the two treatments should be considered as parallel rather than dose-equivalent exposures, and the biological effects of PGJ cannot be attributed solely to its PUN content. Although both treatments influenced several common metabolic regulators, the present experimental design does not establish whether their distinct downstream responses reflect differences in PUN exposure, the contribution of other juice constituents, or interactions within the complex phytochemical matrix. Further concentration-matched investigations would be required to determine the relative contribution of PUN to the biological effects of PGJ.

Previous studies have reported that PUN can activate AMPK signalling and modulate mitochondrial function in mammalian metabolic disease models [48]. In the present study, increased mRNA expression of the AMPK orthologue *aak-2* was observed exclusively following PGJ supplementation, whereas PUN preferentially increased *nhr-49* and related nuclear hormone receptor responses. These findings are consistent with previous reports demonstrating that PUN can modulate lipid-handling enzymes, including lipase, α-glucosidase, and dipeptidyl peptidase-4, thereby influencing metabolic homeostasis [3]. Furthermore, improved insulin sensitivity associated with PUN-mediated enhancement of mitochondrial turnover has been reported in models of palmitate-induced insulin resistance [49], supporting involvement of mitochondrial and lipid-associated processes in PUN-mediated metabolic regulation. Consistent with these observations, both PGJ and PUN increased mitochondrial mass-associated fluorescence and mitochondrial membrane potential-derived fluorescence in *C. elegans*, indicating that both treatments modulate mitochondrial parameters. However, the TMRE/MTG fluorescence ratio was not significantly increased, which indicates unaffected mitochondrial potential. These observations are partially consistent with growing evidence linking ellagitannin-derived metabolites to mitochondrial preserving mechanisms and healthy ageing [50]. In parallel, PGJ and PUN enhanced locomotor activity, including average worm speed, a parameter associated with energy expenditure and metabolic flexibility [51]. This improvement in locomotor performance may therefore be indicative of altered metabolic performance and adaptation [52], consistent with the observed changes in mitochondrial status and conserved metabolic pathways.

The reduction in neutral lipid-associated fluorescence observed under basal conditions further suggests that both PGJ and PUN influence conserved pathways involved in lipid homeostasis. A limitation of the present study is that lipid accumulation was assessed by Nile Red fluorescence, which provides a relative measure of neutral lipid-associated staining rather than direct biochemical quantification of triglyceride content. In the QC155 mutant strain carrying gain-of-function alleles of *nhr-49* and *mdt-15*, neither treatment significantly reduced neutral lipid-associated fluorescence under glucose-enriched conditions. Given the altered lipid homeostasis associated with this genetic background, these findings may reflect differences in the metabolic responsiveness of QC155 animals, although the individual contributions of NHR-49 and MDT-15 remain unresolved. The inclusion of ezetimibe as a pharmacological reference provided a useful benchmark for these effects, as it represents an established lipid-lowering intervention acting through inhibition of intestinal cholesterol absorption. Interestingly, whereas ezetimibe had little effect under basal conditions, its lipid-lowering activity became evident under glucose-enriched conditions, although it remained less pronounced than that observed with PGJ and PUN. Nevertheless, the use of a double gain-of-function mutant does not establish the individual contribution or functional requirement of either regulator.

At the molecular level, PGJ was associated with increased *aak-2* mRNA expression together with selective upregulation of the fatty acid desaturase *fat-6* under basal conditions, whereas under glucose-challenged conditions it additionally increased the expression of *acs-2*, a gene involved in fatty acid utilisation, indicating a broader transcriptional response under metabolic stress. This context-dependent response suggests that PGJ may modulate metabolic pathways according to the prevailing metabolic state rather than through a single constitutively engaged mechanism. These findings are consistent with previous reports demonstrating beneficial effects of pomegranate juice and its bioactive constituents in metabolic disorders, including obesity and type 2 diabetes [35,53].

In parallel, the morphological changes observed in this study, including reduction in body size parameters, may be associated with the decrease in neutral lipid-associated fluorescence, potentially reflecting alterations in lipid storage and metabolic status [54]. However, the relationship between body morphology and neutral lipid-associated fluorescence remains to be established experimentally. Beyond these metabolic effects, both PGJ and PUN enhanced resistance to acute stress conditions, suggesting that their metabolic actions may be accompanied by increased stress resilience. This observation is consistent with previous reports demonstrating that, owing to its antioxidant properties, PUN protects against hypoxia-induced endothelial dysfunction and pulmonary hypertension [55] and attenuates pelvic inflammatory disease in rats [56]. Collectively, these findings support a broader role for PUN in modulating cellular stress responses.

Beneficial effects of PUN were also evident under chronic proteotoxic challenge. In temperature-inducible amyloid-β-expressing *C. elegans*, PUN improved bending frequency, head amplitude, and average velocity, consistent with preserved neuromuscular function observed in wild-type animals. These findings also align with previous studies demonstrating the neuroprotective potential of pomegranate juice and its constituents in experimental models of Alzheimer’s and Parkinson’s diseases [1,5,38,57,58,59]. Notably, PUN treatment also reduced body size parameters in GMC101 animals, resembling the morphological changes observed in wild-type animals. Whether these changes are related to alterations in lipid storage or metabolic status in the GMC101 background, however, remains to be determined. It should also be noted that PGJ was not evaluated in the GMC101 model, and neither Aβ levels nor aggregation were directly assessed. Although PUN significantly improved locomotor performance in this model, the present study cannot establish whether these effects are specific to Aβ-induced proteotoxicity or reflect a broader enhancement of organismal fitness. Future studies comparing wild-type animals exposed to the same temperature-shift protocol, together with analyses of the underlying metabolic regulators in the GMC101 background, will be needed to clarify the specificity and underlying mechanisms of these effects.

At a mechanistic level, emerging evidence implicates MED15 in the regulation of lipid metabolism and cardiovascular disease, owing to its role in coordinating inflammatory signalling and triglyceride homeostasis [60,61]. In *C. elegans*, its orthologue MDT-15 functions as a central regulatory hub integrating metabolic and stress-related signals through interactions with transcriptional regulators such as NHR-49 and SBP-1 [62,63]. Recent studies have highlighted the role of NHR-49/MDT-15 in coordinating lipid metabolism and cellular stress responses, including NHR-49-dependent regulation of the fatty-acid desaturase FAT-7 [64]. In the present study, both PGJ and PUN increased MDT-15 expression, identifying MDT-15 as a shared treatment-responsive component, although the downstream responses differed between the two interventions.

Under basal conditions, PGJ elicited a broader transcriptional response consistent with coordinated remodelling of lipid metabolism, characterised by reduced expression of *sbp-1* and its lipogenic target *pod-2*, together with increased *acs-2* expression and FAT-6 abundance. Although suppression of *sbp-1* would generally be expected to reduce fatty acid desaturation [65], the concomitant increase in FAT-6 suggests that PGJ may engage compensatory or alternative regulatory mechanisms to maintain fatty acid desaturation despite reduced *sbp-1* expression [64]. Such regulation is consistent with the established role of MDT-15 in coordinating lipid synthesis, desaturation and utilisation according to metabolic demand [63,66].

By contrast, PUN treatment elicited a more selective response focused on nuclear hormone receptor signalling, with increased expression of *nhr-49*, *nhr-80*, and *nhr-13*, together with marked induction of FAT-7. This pattern is consistent with the preferential engagement of nuclear hormone receptor-associated lipid remodelling and fatty acid desaturation. Importantly, increased FAT-6 or FAT-7 expression does not necessarily indicate enhanced neutral lipid storage, as these Δ9-desaturases primarily contribute to fatty-acid desaturation and composition. Thus, their increased expression alongside reduced Nile Red-associated fluorescence may reflect alterations in lipid homeostasis rather than increased lipid accumulation. However, the absence of fatty-acid profiling precludes conclusions regarding changes in fatty-acid composition. Overall, PGJ and PUN share MDT-15-associated responses but distinct downstream signatures. PGJ produces broader transcriptional remodelling of lipid metabolism, whereas PUN preferentially engages nuclear hormone receptor-associated pathways. Recent mechanistic studies further support the involvement of NHR-49 and MDT-15 in coordinating lipid metabolism, stress adaptation, and metabolic flexibility in *C. elegans*, including regulation of β-oxidation, fatty acid metabolism, and mitochondrial surveillance [67,68,69]. In parallel, recent studies with bioactive natural compounds have demonstrated NHR-49- and AAK-2-associated modulation of lipid accumulation, including under high-glucose conditions [70,71]. Nevertheless, these findings are limited to *C. elegans* and do not establish direct functional involvement of the proposed regulatory pathways. Further studies in more complex biological models, including mammalian systems, are warranted to validate these molecular responses and assess their broader physiological relevance.

## 4. Materials and Methods

### 4.1. Materials and Reagents

Punicalagin (#HY-N0063, Mw = 1084.72, purity: 99.83%) and Ezetimibe (#HY-17376, Mw = 409.43, purity: 99.88%) were supplied from MedChem Tronica (Sollentuna, Sweden). All solvents utilised in chromatographic analyses were liquid chromatography–mass spectrometry (LC-MS) grade [(H_2_O–LiChrosolv, Sigma-Aldrich Co., St. Louis, MO, USA; Acetonitrile–Chromasolv; Honeywell Intl. Inc., Charlotte, NC, USA; Formic Acid-LiChropur, Sigma-Aldrich Co., St. Louis, MO, USA)]. Deuterated solvents–methanol and water were purchased from Deutero GmbH (Kasbellaun, Germany). The LB broth Lennox (#L3022), agar powder (#05039), M9 minimal salts (#M6030), 3-(4,5-dimethylthiazol-2-yl)-2,5-diphenyl tetrazolium bromide (MTT; #M2128), fluoroshield histology mounting medium (#F6182), Nile red (#72485), paraquat (purity ≥ 98%), were purchased from Sigma-Aldrich (St. Louis, MO, USA). Nematode growth medium (NGM; #MBS652667) was purchased from MyBiosource Inc. (San Diego, CA, USA). Reagents and consumables for RNA isolation, gel electrophoresis and reverse transcription quantitative polymerase chain reaction (RT-qPCR) analyses were supplied by Bio-Rad Laboratories Inc. (Hercules, CA, USA). Tetramethylrhodamine, ethyl ester (TMRE, Cat. No 11560796) was obtained from Invitrogen. MitoTracker Green FM (MTG, #HY-135056) was supplied from MedChem Tronica (Sollentuna, Sweden).

### 4.2. Plant Material and Extraction

Commercially available *P. granatum* fruits were purchased from a local grocery store, allowed to acclimatise at room temperature, and sorted for uniformity, with fruits showing physical defects being discarded. Pomegranate juice was obtained by mechanical pressing of fruits cut in half using a juice extractor. The resulting juice was lyophilized using a VirTis BenchTop Pro freeze dryer with Omnitronics™ controller (SP Industries, Inc. Ipswich, UK). The obtained lyophilized pomegranate juice was stored at −20 °C until chemical and biological analyses. The lyophilized preparation is hereafter referred to as pomegranate juice (PGJ).

### 4.3. Metabolite Profiling Based on Nuclear Magnetic Resonance (NMR) Spectroscopy

The NMR analysis was performed according to the protocol previously described [72]. Briefly, a sample of 10 mg lyophilized pomegranate juice was dissolved in equal amounts of 0.4 mL CD_3_OD and D_2_O, buffered with KH_2_PO_4_ (pH 6.0), and 0.005% (*w*/*v*) 3-(trimethylsilyl)propionic-2,2,3,3-*d*_4_ acid sodium salt (TSPA-*d*_4_) as an internal standard. One-dimensional proton (^1^H) NMR spectrum and two-dimensional spectra–homonuclear correlation spectroscopy (^1^H-^1^H-COSY) and homonuclear total correlation spectroscopy (^1^H-^1^H-TOCSY) spectra were recorded on a Bruker AVII+ 600 spectrometer (Bruker, Karlsruhe, Germany), operating at 25 °C, 600.13 MHz proton frequency, with 4.07 s relaxation time and CD_3_OD as an internal lock. The obtained spectral data were subjected to manual phase and baseline correction, using MestReNova software version 12.0.0 from Mestrelab Research (Santiago de Compostela, Spain). The TSPA-*d*_4_ peak was referenced at 0.0 ppm and used as an internal standard for spectral normalisation to its peak scaled to 1.0. The main metabolites within the pomegranate juice were identified according to comparison with previously reported spectral data.

### 4.4. Liquid Chromatography–Mass Spectrometry Apparatus and Conditions

#### 4.4.1. Apparatus and LC-MS Conditions

Chromatographic analysis was performed by reverse-phase ultra-performance liquid chromatography (UPLC, Waters Corp., Milford, MA, USA) connected to a Synapt XS Ion Mobility Time-of-Flight (qToF) Mass Spectrometer (Waters Corp., Milford, MA, USA). An ACQUITY UPLC HSS T3 column (100 Å, 1.8 µm, 2.1 mm × 100 mm) equipped with an ACQUITY UPLC HSS T3 VanGuard pre-column (100 Å, 1.8 µm, 2.1 mm × 5 mm) was used for separation (Waters Corp., Milford, MA, USA). Samples were eluted at a flow rate of 0.4 mL/min using a multi-step linear gradient with different proportions of Solvent A (H_2_O + 0.1% Formic acid) and Solvent B (Acetonitrile + 0.1% Formic acid) as described: 0–1 min 1% B, 1–11 min 1–40% B, 11–13 min 40–70% B, 13–15 min 70–99% B, 15–16 min 99% B, 16–17 min 99–1% B, 17–20 min 1% B. The injection volume was 1 µL and the column oven was set to 40 °C.

The LC eluent was infused into a Synapt XS Ion Mobility Time-of-Flight (qToF) Mass Spectrometer, and tandem mass spectra data were recorded in MSE acquisition mode. The ionisation mode was negative electrospray (ESI^-^) and the source temperature was set at 120 °C with a desolvation temperature of 250 °C and a desolvation gas flow of 600 L/h. The capillary voltage was set at 2.5 kV with the cone voltage set at 40 V. The collision energy values were set at 4 eV (trap)/2 eV (transfer) for the low collision energy scan and at 20–30 eV ramp (trap)/2 eV (transfer) for the high collision energy scan. The lock mass compound was leucine enkephalin (*m*/*z* 554.2615) (Waters Corp., Milford, MA, USA) in negative ion mode [M-H]^–^ and the range of mass detection was 50–1500 *m*/*z*. Nitrogen was used as the desolvation gas. Punicalagin (PUN, Cat. No.: HY-N0063) standard was used for accurate mass quantification.

#### 4.4.2. Sample and Standards Preparation

Freeze-dried pomegranate juice was dissolved in 50% (*v*/*v*) LC-MS grade methanol (aq) and homogenised using a vortex for 1 min; the supernatant was then centrifuged and filtered through a 0.22 μm PTFE syringe filter into a glass vial for analysis. Three independent sample solutions were prepared from the same lyophilized PGJ and analysed separately. Each sample solution was analysed in triplicate. Quantification was performed using an external standard method. One mg of PUN was dissolved in 1 mL 50% (*v*/*v*) methanol (aq). Standard solutions were prepared in triplicate and from each, serial dilutions ranging from 100 μg/mL to 1.56 μg/mL were prepared for a 7-point calibration curve of PUN. The PUN standard comprises a mixture of both α- and β-isomers, which appear as separate peaks on the extracted ion chromatogram (EIC). For quantification of total PUN, a separate calibration curve for each isomer was constructed by plotting the peak area against the nominal concentration of the standard solution dilutions, corrected according to the α/β isomer ratio provided by the manufacturer.

### 4.5. Caenorhabditis elegans Strains, Maintenance, and Experimental Treatments

The following *C. elegans* strains—wild-type Bristol N2, LD1 *ldIs7* [SKN-1B/C::GFP + *rol-6(su1006)*], MAH240 *sqIs17* [p*_hlh-30_*HLH-30::GFP + *rol-6(su1006)*], IJ1651 *mdt-15(yh44*[*mdt-15*::degron::EmGFP]) III, CL2166 *dvIs19* [(pAF15)p*_gst-4_*GFP::NLS] III, AGD467 *uthEx490* [*aak-2* (intron 1)::AAK-2(aa1–aa321 T172D)::GFP + p*_crtc-1_*CRTC-1::tdTomato + *rol-6(su1006)*], DMS303 *nIs590* [p*_fat-7_*FAT-7::GFP + *lin-15(+)*] V, VS20 *hjIs67* [p*_atgl-1_*ATGL-1::GFP + MEC-7::RFP], PHX649 *fat-6(syb649*[FAT-6::GFP]) IV, PHX3258 *nhr-49(syb3258*[NHR-49::GFP]) I, QC155 *nhr-49(et8) I*; *mdt-15(et14)* III, and GMC101 *dvIs100* [p*_unc-54_*A-beta-1-42::unc-54 3′-UTR + p*_mtl-2_*GFP], were maintained under standard conditions at 20 °C on nematode growth medium (NGM) plates seeded with *Escherichia coli* OP50. All strains were provided by the Caenorhabditis Genetics Center (CGC, University of Minnesota, Minneapolis, MN, USA), which is funded by the NIH Office of Research Infrastructure Programs (P40 OD010440).

For subsequent experiments, age-synchronised populations of *C. elegans* were obtained using a standard hypochlorite bleaching technique as previously described [13]. Briefly, worms were collected in M9 buffer (up to 3 mL), followed by treatment with an alkaline hypochlorite solution containing sodium hydroxide (5 N) and household bleach (5% hypochlorite). The suspension was then vortexed to facilitate the disruption of adult worms, followed by centrifugation. After removal of the supernatant, the resulting egg pellet was washed with distilled water to eliminate residual bleaching solution and centrifuged again.

The PGJ and PUN were dissolved in DMSO (0.4%), and the stock solutions were subsequently added to heat-inactivated, tenfold-concentrated *E. coli* OP50 to achieve the final experimental concentrations. For the evaluation of lipid dynamics, glucose was added to the NGM to a final concentration of 2%.

The PGJ was tested at 50, 100, and 200 μg/mL, whereas PUN was tested at 25, 50, and 100 μM, based on the respective tolerability profiles determined in the viability assay described in Section 4.6. The concentrations of PGJ and PUN were selected independently based on their respective tolerability profiles, as these treatments were intended to characterise the biological responses to the complex juice matrix and PUN rather than to provide equivalent PUN exposure. A vehicle group containing 0.4% DMSO was used as a control. Ezetimibe (EZ; 10 μM), a clinically established inhibitor of intestinal cholesterol absorption, was included as a pharmacological reference compound in the lipid-accumulation assays. EZ was selected to provide a clinically relevant benchmark for comparison with the lipid-modulating effects of PGJ and PUN and to assess whether these treatments produce metabolic responses comparable to those elicited by an established lipid-lowering intervention [29,30].

### 4.6. Viability Assessment

Viability was assessed as previously described using the 3-(4,5-dimethylthiazol-2-yl)-2,5-diphenyl tetrazolium bromide (MTT) assay [13]. Briefly, worms (L1 larval stage) were pre-treated with PGJ (10, 50, 100, 200 μg/mL), PUN (25, 50, 100, 250 μM) or Vehicle (0.4% DMSO) for 48 h, washed with M9 buffer, and incubated with MTT solution (5 mg/mL) for 3 h. After formazan crystal formation, the reagent was removed, and samples were incubated with DMSO for 1 h to ensure complete solubilisation.

### 4.7. Fecundity Assay

To investigate the effect of PGJ (50, 100, 200 μg/mL) on the reproductive function and egg-laying capacity of *C. elegans*, fecundity was assessed by counting the daily and total progeny according to the previously described standard procedure [13].

### 4.8. Locomotion Assay

Worms pre-treated with PGJ (50, 100, 200 μg/mL) for 5 or 10 days were randomly selected and placed in a drop of M9 buffer in accordance with a previously described procedure [32]. Following a 30 s acclimatisation period, their bending movements were monitored over a 30 s interval. For each experimental group, at least 15 worms were analysed in three independent experiments (n = 45).

### 4.9. Lifespan Measurement

Lifespan was performed according to the standard protocol previously described [13] in detail. For each experimental group, at least 90 worms from three independent replicates were analysed. Worms lost because of crawling off the plate, internal hatching or accidental injury were treated as censored observations. Details on the mean and maximum lifespans under PGJ (50, 100, 200 μg/mL) treatment are presented in Supplementary Table S2.

### 4.10. Thermotolerance Assay

Heat stress resilience assay was performed using a minimum of 90 animals per group, and each experiment was conducted independently at least three times. Synchronised populations of N2 worms (n ≥ 100) at the late L4 larval stage were cultured under standard conditions [13] on NGM plates, with or without PGJ (50, 100, 200 μg/mL), PUN (25, 50, 100 μM), or Vehicle (0.4% DMSO). Treatment continued until the worms reached 5 or 10 days of adulthood. At each time point, nematodes from each experimental group were randomly selected and incubated at 37 °C for 2 h, followed by a 20 h recovery period at 20 °C. Survival was subsequently assessed by gently touching the worms with a platinum wire.

### 4.11. Oxidative Stress Assay

To assess oxidative stress resistance, synchronised populations of a minimum of 150 nematodes per group (n ≥ 100) were supplemented with PGJ (50, 100, 200 μg/mL), PUN (25, 50, 100 μM) or Vehicle (0.4% DMSO). Upon reaching the 5th and 10th days of their lifespan, at least 90 worms per group were randomly selected and transferred to fresh NGM plates containing 50 mM paraquat using the procedure described earlier [13]. The number of deceased individuals was recorded at 24 h intervals until the last worm had died. Details on the mean and maximum survival under the respective treatments are presented in Supplementary Table S3.

### 4.12. Analysis of Body Morphology

The analysis of body morphology, including mean body length, width, and area, was conducted by the automated imaging, tracking, and quantitative analysis system–WormLab (MBF Bioscience, Williston, VT, USA) as previously described [54]. Worms were pretreated with PGJ (50, 100, 200 μg/mL), PUN (25, 50, 100 μM) or vehicle (0.4% DMSO) for 24 h, after which a 1 min video was recorded for each group and analysed using the WormLab software (version 2023.1.1, MBF Bioscience, Williston, VT, USA) as described earlier [73].

### 4.13. Nile Red Lipid Staining

Nile red lipid staining for both N2 and QC155 strains was performed as described previously in detail [13]. Briefly, after obtaining a synchronised population, worms in each group were exposed to either glucose (2%) or normal conditions along with the corresponding experimental treatments. Approximately 500 worms (late L4 larval stage) per sample were then fixed in 40% isopropyl alcohol for 3 min, then worms were stained with Nile Red solution for 2 h. After staining, the worms were washed and mounted on microscope slides. Imaging was performed using the Stellaris 5 laser confocal system coupled to an inverted DMi8 microscope (Leica, Wetzlar, Germany). Images for analysis were acquired at 10×, while representative images were at 20× magnification. Within each experiment, laser power, detection gain, pinhole, and acquisition settings were maintained constant across vehicle- and treatment-exposed groups. Fluorescence intensity was quantified using ImageJ software (version 1.53t, NIH, Bethesda, MA, USA) and normalised to the control group. Results are reported as normalised corrected total cell fluorescence (CTCF).

### 4.14. Mitochondrial Co-Staining

For evaluation of the mitochondrial mass and membrane-potential-associated fluorescence, after pre-treatment with PGJ (200 μg/mL), PUN (100 μM) or vehicle (0.4% DMSO), at least 100 worms from each experimental group were co-stained with MTG (4 µM) and TMRE (100 µM) for 24 h, at a final concentration in the food source. On the following day, the nematodes were subjected to a destaining procedure for 1 h, followed by mounting and preparation of microscopic preparations.

For each experimental group, more than 60 nematodes were randomly selected for analysis. Image acquisition of the worms (late L4 larval stage) was subsequently performed using a Stellaris 5 confocal system coupled with an inverted DMi8 microscope (Leica). The MTG and TMRE fluorescence was acquired sequentially using the corresponding fluorescence channels, with identical acquisition settings maintained across experimental groups. Quantification of fluorescence intensity was performed using ImageJ software (version 1.53t) as earlier described [13]. The data are reported as normalised to the vehicle group CTCF.

### 4.15. Confocal Imaging of Transgenic Reporter Strains

The imaging of the following reporter and mutant strains–LD1, MAH240, IJ1651, CL2166, AGD467, DMS303, VS20, PHX649, and PHX3258–was assessed in accordance with standard procedures described earlier [13,74,75,76,77]. For each experimental group more than 60 nematodes were randomly selected for analysis. Fluorescence signals from GFP- or tdTomato-tagged reporters were acquired at 10× magnification using appropriate fluorescence filter sets on a Leica Stellaris 5 confocal system coupled with an inverted DMi8 microscope (Leica). Images for analysis were acquired at 10×, while representative images–at 20× magnification. For each reporter strain, laser power, detection gain, pinhole, and acquisition settings were maintained constant across vehicle- and treatment-exposed groups. For the LD1 strain, fluorescence intensity was quantified exclusively in the ASI neurons. Quantification of FAT-7::GFP expression in the DMS303 strain was performed as the fraction of GFP-positive animals, in accordance with [75]. For FAT-6::GFP in the PHX649 strain, fluorescence intensity was quantified as the mean grey value within intestinal regions of interest (ROIs) after background subtraction and expressed as normalised corrected mean fluorescence (CMF). Fluorescence signals from all other reporter strains were expressed as CTCF relative to the vehicle-treated control group [13,74]. The fluorescence quantification of the other strains were performed using ImageJ software (version 1.53t) and represented as normalised to the vehicle group CTCF.

### 4.16. Gene Expression Analysis by RT-qPCR

A synchronised population of approximately 3000–4000 L4 nematodes per treatment group was exposed to vehicle (0.4% DMSO), PGJ (50, 100, 200 μg/mL), or PUN (25, 50, 100 μM) for 24 h at 20 °C. Total RNA extraction, assessment of RNA integrity and quantity, cDNA synthesis, and qPCR reactions were conducted according to the protocols previously described [13]. For qPCR analysis, *iscu-1* and *pmp-3* were used as reference genes to normalise the expression levels of target genes. Three independent biological experiments were performed, each measured in technical triplicate. The nucleotide sequences of the primers used are detailed in Supplementary Table S4.

### 4.17. In Vivo Assays Using SydLab™ One in C. elegans Alzheimer’s Disease Model

Experiments were performed using the *C. elegans* transgenic strain GMC101, a temperature-inducible Alzheimer’s disease model expressing human amyloid-β (Aβ1–42) in body wall muscle cells. Age-synchronised populations were loaded into microfluidic chips and maintained for 15 days under fully automated environmental control using the SydLab™ One platform (Nagi Bioscience SA, Saint-Sulpice, Switzerland). To induce amyloid-β expression, animals were exposed to 25 °C for 48 h. Following induction, worms were returned to and maintained at 20 °C for the remainder of the assay. Punicalagin at 100 μM concentration was administered from start to end during the length of the assay, along with the vehicle group.

Following the assay, phenotypic and healthspan parameters were calculated. Survival was quantified using the standard SydLab™ One analysis survival analysis software under patent # EP4137558A1 (Nagi Bioscience SA). In brief, high-content imaging data were continuously acquired, and live versus dead animals were automatically classified using SydLab™ One machine-learning algorithms based on motility and morphological features. Behavioural and locomotor parameters were quantified using standardised SydLab™ One worm metric analysis software (Nagi Bioscience SA). Extracted endpoints included worm area, bending frequency, head amplitude, average velocity, and body area, providing quantitative measures of neuromuscular function and worm metric parameters following amyloid-β induction.

### 4.18. Statistical Analysis

Statistical analyses were performed using SigmaPlot v11.0 (Systat Software GmbH, Erkrath, Germany). Data are presented as mean ± SEM unless otherwise specified. Comparisons among multiple experimental groups were performed using one-way analysis of variance (ANOVA), followed by Tukey’s post hoc test. Normality of the data was assessed using the Shapiro–Wilk test. For the datasets that did not satisfy the normality assumption, ANOVA on ranks (Kruskal–Wallis test), followed by Dunn’s multiple-comparison test, was applied. Comparisons between two groups were performed using Student’s *t*-test when appropriate.

Effect sizes were calculated for the principal statistical comparisons. Eta-squared (η^2^) was used as the effect-size measure for one-way ANOVA, whereas epsilon-squared (ε^2^) was calculated for Kruskal–Wallis analyses. Relative or fold changes compared with the corresponding vehicle group are additionally reported to indicate the magnitude of treatment effects. For selected key quantitative outcomes, 95% confidence intervals (95% Cis) of group means were additionally reported as descriptive estimates.

Lifespan and oxidative stress survival data were analysed using Kaplan–Meier survival curves, and differences between groups were evaluated using the log-rank test. The number of animals analysed is shown. Statistical significance was set at *p* < 0.05 with significance levels indicated as * *p* < 0.05, ** *p* < 0.01, *** *p* < 0.001, **** *p* < 0.0001 compared with the vehicle group. All experimental data shown are representative of at least three independent biological experiments.

## Data Availability

The original contributions presented in this study are included in the article and Supplementary Materials. Further inquiries can be directed to the corresponding author.

## Supplementary Materials

The following supporting information can be downloaded at https://www.mdpi.com/article/10.3390/ijms27219852/s1.
