# Supplementary material for: Metabolic Rewiring by Pomegranate Juice and Punicalagin Is Associated with NHR-49/MDT-15-Related Responses and Enhanced Stress Resilience
Source: Int J Mol Sci. 2026 Nov 3;27(21):9852. doi: 10.3390/ijms27219852 (PMC13763278; doi:10.3390/ijms27219852)
Supplement: Supplementary file 1 [file ijms-27-09852-s001.zip › ijms-4618008-supplementary.pdf]

Supplementary Information

Metabolic rewiring by pomegranate juice and punicalagin is associated with NHR-49/MDT-15-related responses and enhanced stress resilience

Monika N. Todorova[1,2], Biser K. Binev[1,2], Christina Ploumi[3], Eleni Tsakiri[3], Antonis Roussos[3], Dimitre A. Ivanov[4,5], Martina S. Savova[1,2]*, Konstantinos Palikaras[3], Milen I. Georgiev[1,2]

[1]Laboratory of Metabolomics, Institute of Microbiology, Bulgarian Academy of Sciences, 4000 Plovdiv, Bulgaria
[2]Department of Plant Cell Biotechnology, Center of Plant Systems Biology and Biotechnology, 4023 Plovdiv, Bulgaria
[3]Department of Physiology, Medical School, National and Kapodistrian University of Athens, Athens, Greece
[4]Department of Plant Metabolomics, Center of Plant Systems Biology and Biotechnology, 4023 Plovdiv, Bulgaria
[5]Department of Biology, University of Western Ontario, London, ON, N6A 5B7, Canada

*Corresponding author: Martina S. Savova, Laboratory of Metabolomics, Department of Biotechnology, Institute of Microbiology, Bulgarian Academy of Sciences, 139 Ruski Blvd., 4000 Plovdiv, Bulgaria; e-mail: m.sav@abv.bg; tel: +359 32 64 24 30;

Supplementary Figures

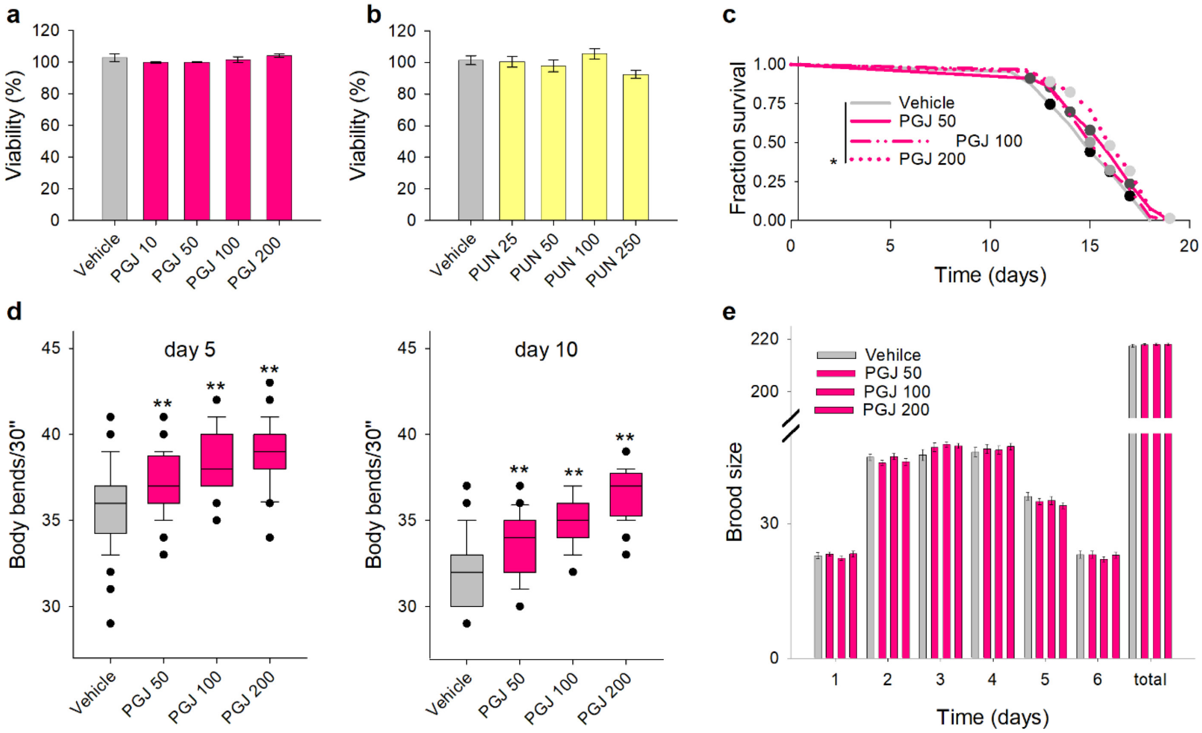

Figure S1. Pomegranate juice (PGJ) and punicalagin (PUN) effect on healthspan parameters. Viability assessment using MTT assay upon (a) PGJ (10–200 μg/mL), and (b) PUN (25–250 μM) in *C. elegans* (n = 1500) compared to control group. (c) Fraction survival of PGJ-supplemented nematodes, compared to vehicle group, represented as a Kaplan–Meier survival curve (n = 90), *p<0.05. (d) Locomotion of the PGJ-fed nematodes (50, 100, and 200 μg/mL) on day 5 and day 10, presented as number of body bends in 30 sec in comparison to the vehicle group (n = 45), *p<0.01. (e) Daily and total brood size of PGJ-supplemented nematodes (n = 15) For each group, n consisted of a total number of worms per treatment group from at least three independent biological replicates.

Supplementary Tables

Table S1. Compounds determined using NMR spectroscopy in *P. granatum* juice. Chemical shifts (δ) and coupling constants (J) identified by their relevant ^{1}H and 2D COSY and TOCSY NMR spectra compared with the literature [18–23] or available spectral databases [24,25].

	Metabolite	^{1}H δ, ppm (J, Hz)	^{1}H-^{1}H COSY ($\delta_H \leftrightarrow \delta_H$)	^{1}H-^{1}H TOCSY ($\delta_H \leftrightarrow \delta_H$)
Sugars	α-Glucose	5.18 (d, J = 3.71); 3.46 (dd, J = 9.76, J = 3.77)	5.18↔3.46	5.18↔3.46
	β-Glucose	4.57 (d, J = 7.96); 3.19 (dd, J = 9.33, J = 8.01)	4.57↔3.19	4.57↔3.19
	Fructose	4.02 (dd, J = 12.72, J =1.20),	–	–
	Sucrose	4.16 (d, J = 8.65); 5.40 (d, J = 3.89)	–	–
	Arabinose	5.31 (d, J = 3.64)	–	–
Amino acids	Alanine	1.48 (d, J = 7.24); 3.69 (m)*	1.48↔3.69	1.48↔3.69
	Isoleucine	0.87 (t, J = 7.27)	–	–
	Valine	1.00 (d, J = 7.01); 1.05 (d, J = 7.08)	–	–
	Threonine	1.33 (d, J = 6.8); 3.49 (m); 4.2 (s)	–	1.33↔4.2
	Glutamine	2.13 (m); 2.44 (m); 3.7 (s)	2.13↔2.44	2.13↔2.44; 2.13↔3.7; 2.44↔3.7
	γ-Amino-butyrate (GABA)	1.92 (m); 2.34 (t, J = 7.5); 3.01 (t, J = 7.4)	1.91↔3.01; 1.91↔2.34	–
Organic acids	Citric acid (CA)	2.69 (d, J = 15.5); 2.78 (d, J = 15.5)	2.69↔2.78	–
	Malic acid (MA)	2.52 (dd, J = 15.83, 8.45); 2.78 (d, J = 15.57); 4.30 (dd, J = 8.42, 4.32)	2.78↔4.30; 2.52↔4.30; 2.52↔2.78	2.78↔4.30; 2.52↔4.30; 2.52↔2.78
Ellagitannins	Ellagic acid	7.08 (s)	–	–
	α-/β-Punicalagin	7.08, 6.97, 6.96, 6.87, 6.83, 6.75, 6.69, 6.64	–	–

*-overlapping spectral region;

Table S2. Mean and maximum lifespan (in days) of *C. elegans* upon supplementation with *P. granatum* juice (PGJ, 50, 100, and 200 μg/mL).

Strain	Experimental group	Mean lifespan (SEM)	Maximum lifespan (SEM)
N2 (wild type)	Vehicle	15.00 (0.22)	18.00 (0)
	PGJ 50	15.66 (0.22)	18.7 (0.17)
	PGJ 100	15.45 (0.20)	18.2 (0.15)
	PGJ 200	16.15 (0.20)	18.56 (0.18)

Table S3. Pomegranate juice (PGJ, 50, 100, and 200 μg/mL) and punicalagin (PUN, 25, 50, and 100 μM) increased mean and maximum survival time upon acute oxidative stress (50 mM paraquat) in *C. elegans* on day 5 and 10 of adulthood.

Time point	Experimental group	Mean survival (SEM)	Maximum survival (SEM)
day 5	Vehicle	2.11 (0.10)	3.11 (0.11)
	PGJ 50	2.6 (0.12)	4.33 (0.16)
	PGJ 100	2.86 (0.13)	5 (0)
	PGJ 200	3.52 (0.15)	5.67 (0.16)
day 10	Vehicle	1.42 (0.07)	3 (0)
	PGJ 50	1.69 (0.09)	3.11 (0.11)
	PGJ 100	2.11 (0.13)	4.67 (0.17)
	PGJ 200	2.46 (0.15)	5.11 (0.20)
day 5	Vehicle	2.18 (0.12)	4 (0)
	PUN 25	2.24 (0.12)	4 (0)
	PUN 50	2.46 (0.13)	4.44 (0.18)
	PUN 100	2.88 (0.14)	5 (0)
day 10	Vehicle	1.58 (0.08)	3 (0)
	PUN 25	1.64 (0.09)	3 (0)
	PUN 50	1.67 (0.09)	3 (0)
	PUN 100	2.37 (0.11)	4 (0)

Table S4. Primer sequences used in the RT-qPCR of mRNAs.

Gene	Forward (5′ → 3′)	Reverse (5′ → 3′)
aak-2	GCCATTTGATGATGAGCACGTG	TACGCAGAGCATATGGTGAAGC
acs-2	AGTTGGACTATGTCGCTGATGC	TCTTTCCTTCATCCTCAACGCG
fat-6	ACTTCCATCACACATTCCCACA	TCGTAGACAAGTCCAAGAGCAG
fat-7	GTTTCACACTTCACGCCACTTG	CCAACGGCTACAACTGTGGTAA
hlh-30	TGAACGCCGACGAAGATATAAT	GTAGTCACACGAGGCTTTTAGA
iscu-1	CCAAAGAATTGTGTCTTCCACC	GCCTTCTTAGTCTGCTTCTTCT
mdt-15	TGTGTTCGCAAAGTGTATGTCG	ACTGTGATGGTTGTAACACGGA
nhr-13	CTGATCCGGGCTCTTCATCATG	ACGTGGCGAATGTTCACCATAA
nhr-49	TCCGAGTTCATTCTCGACG	GGATGAATTGCCAATGGAGC
nhr-80	TATGGAGACAGAAGCCAGGGAG	CAAGCTTTGCCTGACCCAAAAG
pmp-3	TACTTGATCGAGAGCAGTTGCG	CTCTTCTTTCGGAGGTTCTGGC
pod-2	CTTTCAGCGTTGTTCGGATTCG	TGGAGCAACTTTCCAGTGTACG
sbp-1	GATGCGAAAAGGTTGGAGCATG	CTTTCAGCGTTGTTCGGATTCG
